# Repurposing primaquine diphosphate for imatinib-resistant chronic myeloid leukemia via targeting BCR-ABL and Wnt/β-catenin pathway

**DOI:** 10.1016/j.isci.2026.116415

**Published:** 2026-06-30

**Authors:** Changqing Yin, Wenhao Liu, Chensheng Ma, Zhida Zhang, Zenghui Fang, Jiawei Cao, Guang Wu, Haihua Gu, Licai He

**Affiliations:** 1 Key Laboratory of Laboratory Medicine, Ministry of Education, Wenzhou Key Laboratory of Cancer Pathogenesis and Translation, School of Laboratory Medical and Life Science, Wenzhou Medical University, Wenzhou 325035, China; 2 Department of Clinical Laboratory, Affiliated Jinhua Hospital, Zhejiang University School of Medicine, Jinhua, China

**Keywords:** cell biology, cancer

## Abstract

Chronic myeloid leukemia (CML) is defined by the Philadelphia chromosome-derived *BCR::ABL1* fusion gene. Imatinib, a first-line treatment targeting the BCR-ABL protein, has markedly improved clinical outcomes in patients with *BCR::ABL1*^*+*^ CML. However, drug resistance remains a critical therapeutic challenge in the management of CML. In this study, we uncovered that low concentrations of antimalarial drug primaquine diphosphate (PRQ) induced the differentiation of imatinib-resistant CML cells, while high concentrations triggered their apoptosis. Mechanistically, PRQ inhibited the Wnt/β-catenin pathway and promoted the degradation of both wild-type and multiple imatinib-resistant *BCR::ABL1* mutants. Furthermore, PRQ effectively induced differentiation, inhibited colony formation of primary CML blasts, and suppressed the neoplastic growth of imatinib-resistant CML cells in a murine xenograft model. These findings suggest that the antimalarial drug PRQ may have potential for overcoming imatinib resistance in CML, warranting further evaluation as a repurposed candidate.

## Highlights

• PRQ induces differentiation or apoptosis of imatinib-resistant CML cells
• PRQ degrades wild-type and mutant BCR-ABL protein
• PRQ inhibits Wnt/β-catenin signaling pathway
• PRQ suppresses the colony formation of primary CML blasts and xenograft tumorgrowth

## Graphical abstract

**Figure:**
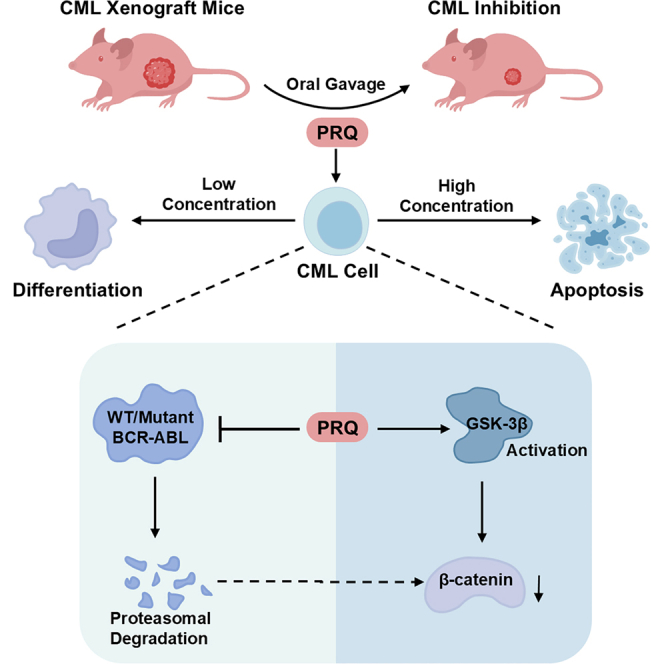

## Introduction

Chronic myeloid leukemia (CML) is a myeloproliferative neoplasm originating from hematopoietic stem cells, which exhibits dysregulated proliferation and impaired differentiation/maturation of myeloid lineages. CML is primarily defined by the presence of Philadelphia chromosome (Ph) as a genetic feature, driven by the t (9; 22) chromosomal translocation, and leading to the production of *BCR::ABL1* fusion gene and its oncogenic protein product, BCR-ABL.^1^^,^^2^ The constitutive tyrosine kinase activity of BCR-ABL drives uncontrolled proliferation and survival of leukemic cells, constituting the molecular basis of CML pathogenesis.^3^ Tyrosine kinase inhibitors (TKIs) targeting BCR-ABL have dramatically improved the overall survival of patients with *BCR::ABL1*^+^ CML.^4^ Imatinib, the first generation of TKI, serves as a first-line drug for the treatment of CML, which exhibits a high cytological response rate.^5^^,^^6^^,^^7^

However, more than 25% of patients with CML develop primary or secondary imatinib resistance.^8^^,^^9^ Point mutations of *BCR::ABL1*-induced drug resistance has become a major therapeutic challenge in CML management. The new generations of TKIs, including dasatinib and ponatinib, provide more treatment options for patients with CML harboring *BCR::ABL1* mutations.^10^ Nevertheless, accumulating clinical evidence reveals that new generations of TKI are associated with serious cumulative toxicity in clinical practice, including vascular occlusive events and pulmonary hypertension.^11^^,^^12^ Studies have shown that some natural molecules can exert anti-leukemic effects through multi-targeted mechanisms.^13^ Consequently, alternative therapeutic strategies for CML are required to overcome imatinib resistance.

Primaquine diphosphate (PRQ) is an established antimalarial agent that interferes with plasmodial mitochondrial function to prevent malaria recurrence.^14^^,^^15^ Our previous study demonstrated the inhibitory efficacy of PRQ against acute myeloid leukemia cells.^16^ It also exhibited an inhibitory effect on breast cancer cells.^17^ This work established PRQ as a dual-target therapeutic drug for imatinib-resistant CML, simultaneously inducing cellular differentiation and apoptosis in imatinib-resistant CML cells. Our mechanistic studies demonstrated that PRQ inhibited the Wnt/β-catenin signaling pathway and facilitated the degradation of BCR-ABL proteins, including both wild-type and various imatinib-resistant mutants. Furthermore, PRQ induced differentiation and suppressed colony formation of CML primary blasts, as well as suppressed the neoplastic growth of imatinib-resistant CML cells in murine xenograft models. These results suggest the therapeutic potential of PRQ for overcoming imatinib resistance in CML.

## Results

### PRQ exhibits an inhibitory effect on the growth of CML cells

**Figure 1:**
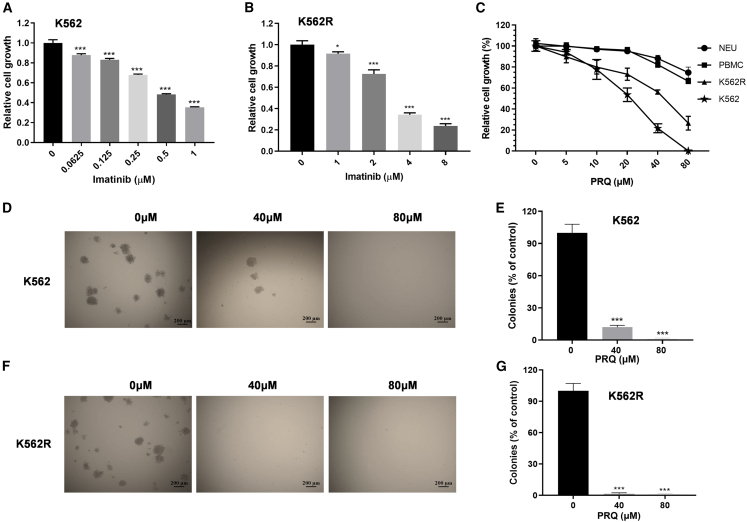
PRQ exhibits an inhibitory effect on the growth of CML cells (A) K562 cells were treated with 0, 0.0625, 0.125, 0.25, 0.5, and 1 μM imatinib for 48 h, and the growth of K562 cells was examined using the CCK-8 assay (*n* = 3). (B) Imatinib-resistant K562R cells were established by inducing drug resistance in K562 cells through gradually increasing the concentration of imatinib. K562R cells were treated with 0, 1, 2, 4, and 8 μM imatinib for 48 h, and the growth of K562R cells was examined using the CCK-8 assay (*n* = 3). (C) K562, K562R, normal neutrophils, and PBMCs were treated with 0, 5, 10, 20, 40, and 80 μM PRQ for 48 h. The inhibitory effect on cell growth was examined using CCK-8 assay (*n* = 3). (D) K562 cells were cultured in methylcellulose-based media containing PRQ (0, 40, and 80 μM). Colonies were counted two weeks after plating, and representative images of colonies were shown (40×). Scale bars, 200 μm. (E) The values correspond to the percentage inhibition of colony formation relative to the vehicle control for K562 cells (*n* = 3). (F) K562R cells were cultured in methylcellulose-based media containing PRQ (0, 40, and 80 μM). Colonies were quantified following two-week incubation, with representative photomicrographs to show morphological differences of colonies (40×). Scale bars, 200 μm. (G)The values correspond to the percentage inhibition of colony formation relative to the vehicle control for K562R cells (*n* = 3). Data are presented as mean ± SD. One-way ANOVA; ∗*p* < 0.05 and ∗∗∗*p* < 0.001versus the control group.

K562R cells were established by gradually increasing imatinib concentrations in K562 cells to induce drug resistance (Figures 1A and 1B). The IC50 of imatinib for K562R cells was 10.59-fold higher than that for parental K562 cells, confirming the resistant phenotype. PRQ inhibited the growth of both K562 and K562R cells, whereas it had minimal effects on normal neutrophils and peripheral blood mononuclear cells (PBMCs) (Figure 1C). Furthermore, treatment with PRQ significantly inhibited the clonogenic growth of CML cells in semisolid methylcellulose cultures, reducing the number of colonies of both K562 (Figures 1D and 1E) and K562R cells (Figures 1F and 1G).

### PRQ induces differentiation and apoptosis of imatinib-resistant CML cells

**Figure 2:**
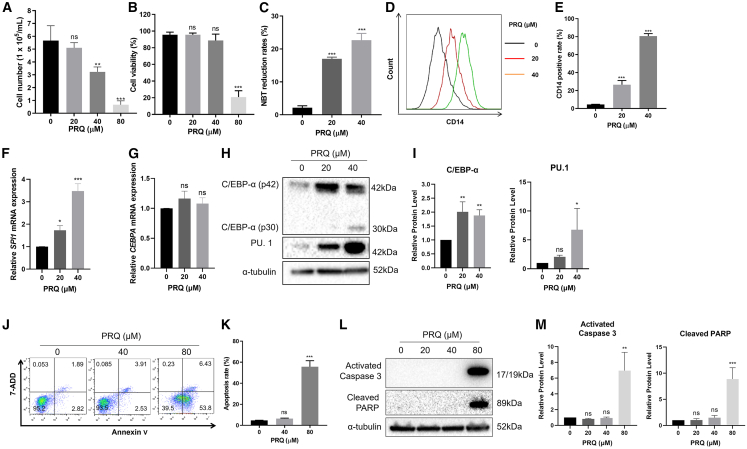
PRQ induces the differentiation and apoptosis of imatinib-resistant CML cells (A and B) K562R cells were exposed to increasing concentrations of PRQ (0, 20, 40, and 80 μM) for 48 h. Viable cell numbers and the cell viability of K562R cells were determined using the trypan blue exclusion assay (*n* = 3). (C) K562R cells were exposed to increasing concentrations of PRQ (0, 20, and 40 μM) for 48 h. The corresponding NBT reduction rates were quantified (*n* = 3). (D and E) K562R cells were exposed to increasing concentrations of PRQ (0, 20, and 40 μM) for 48 h. The percentage of CD14^+^ cells was detected by flow cytometry (*n* = 3). (F and G) K562R cells were treated with 0, 20, and 40 μM PRQ for 48 h, and the mRNA expression of *SPI1* and *CEBPA* was quantified using qPCR (*n* = 3). (H and I) K562R cells were exposed to increasing concentrations of PRQ (0, 20, and 40 μM) for 48 h. Cell proteins were subjected to immunoblotting with the indicated antibodies and quantitatively analyzed (*n* = 3). (J and K) K562R cells were exposed to increasing concentrations of PRQ (0, 40, and 80 μM) for 48 h, and flow cytometry was used to detect the percentage of apoptotic cells using Annexin-V/7-AAD staining assay (*n* = 4). (L and M) K562R cells were exposed to increasing concentrations of PRQ (0, 20, 40, and 80 μM) for 48 h. Cell proteins were immunoblotted with the indicated antibodies and quantitatively analyzed (*n* = 3). Data are presented as mean ± SD. One-way ANOVA; ns (not significant), ∗*p* < 0.05, ∗∗*p* < 0.01, and ∗∗∗*p* < 0.001 versus the control group.

To further investigate the growth-inhibitory effects of PRQ on K562R cells, cell viability was assessed using the trypan blue exclusion assay. The results showed that 40 and 80 μM PRQ decreased the number of viable K562R cells in a concentration-dependent manner (Figure 2A). Notably, lower concentrations of PRQ (20 and 40 μM) did not change the viability of K562R cells, whereas a higher concentration (80 μM) of PRQ significantly decreased cell viability (Figure 2B).

Based on these findings, the effect of low concentration of PRQ on the differentiation of CML cells was examined. PRQ treatment caused a significant increase in NBT reduction, an indicator of myeloid cell functional maturation (Figures 2C and S1A). Lower concentrations of PRQ also induced a marked expansion of the CD14-positive cell population (monocytic marker) in a manner dependent on concentration. (Figures 2D and 2E). To investigate the underlying mechanism, we examined the expression of the master transcription factors PU.1 and C/EBPα.^18^^,^^19^ PRQ treatment significantly increased *SPI1* (encoding PU.1) mRNA levels in a dose-dependent manner (Figure 2F), while *CEBPA* mRNA levels remained unchanged (Figure 2G). Western blot analysis revealed that both PU.1 and C/EBPα protein levels were upregulated (Figures 2H, 2I, S1B, and S1C). To further substantiate monocytic differentiation, we examined additional lineage-specific markers. As shown in Figures S2A–S2D, PRQ did not alter CD235a (erythroid marker) or CD15 (granulocytic marker) expression. These results confirm that PRQ specifically induces the monocytic differentiation of K562 cells. These data indicate that lower concentrations of PRQ upregulate PU.1 at the transcriptional level and C/EBPα at the translational level to induce the monocytic differentiation of CML cells.

A higher concentration of PRQ (80 μM) significantly decreased K562R cell viability, suggesting the induction of cell death. To investigate this, the effect of 80 μM PRQ on the apoptosis of K562R cells was assessed. Flow cytometry analysis indicated that 80 μM PRQ significantly induced apoptosis (Figures 2J, 2K, S3A, and S3B). In addition, 80 μM PRQ treatment activated caspase-3, leading to the cleavage of its substrate PARP (Figures 2L, 2M, S3C, and S3D). These data demonstrated that higher concentrations of PRQ induced apoptosis of CML cells. PRQ exhibits dual anti-proliferative activity through enhancing differentiation and apoptosis depending on its concentrations.

### PRQ inhibits the wnt/β-catenin signaling pathway and induces BCR-ABL protein degradation

**Figure 3:**
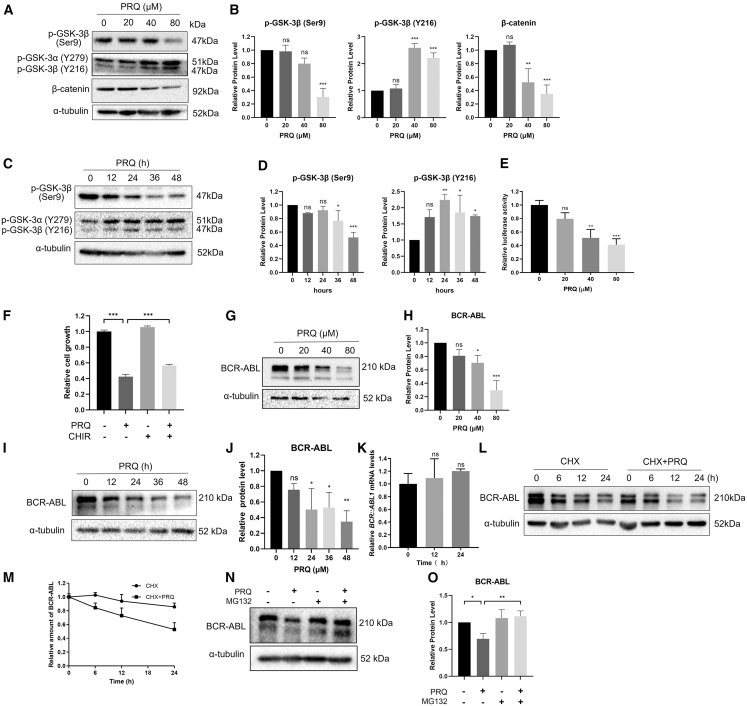
PRQ inhibits the Wnt/β-catenin signaling pathway and induces BCR-ABL protein degradation (A and B) K562R cells were exposed to increasing concentrations of PRQ (0, 20, 40, and 80 μM) for 48 h. Cell proteins were subjected to immunoblotting with the indicated antibodies and quantitatively analyzed (*n* = 3). The anti-phospho-GSK-3α (Tyr279) antibody recognizes both *p*-GSK-3α (Tyr279) and *p*-GSK-3β (Tyr216) due to sequence homology. *p*-GSK-3α at ∼51 kDa (upper band) and *p*-GSK-3β at ∼47 kDa (lower band). (C and D) K562R cells treated with 80 μM PRQ for 0, 12, 24, 36, and 48 h were subjected to immunoblotting with the indicated antibodies and quantitatively analyzed (*n* = 3). (E) 293T/17 cells cotransfected with TOP flash-luciferase (firefly) reporter and TK-renilla luciferase plasmids were treated with 0, 20, 40, and 80 μM PRQ for 48 h. The luciferase activities were normalized against TK-renilla activities (*n* = 3). (F) K562R cells were treated with or without CHIR-99021 (1 μM) and/or PRQ (40 μM) for 48 h. Relative cell growth was analyzed (*n* = 3). (G and H) K562R cells treated with 0, 20, 40, and 80 μM PRQ for 48 h were subjected to immunoblotting with the indicated antibodies and quantitatively analyzed (*n* = 3). (I and J) K562R cells treated with 40 μM PRQ for 0, 12, 24, 36, and 48 h were immunoblotted with the indicated antibodies and quantitatively analyzed (*n* = 3). (K) K562R cells were treated with 40 μM PRQ for 0, 12, and 24 h, and the expression of *BCR::ABL1* mRNA was quantified using qPCR (*n* = 3). (L and M) K562R cells were treated in the presence of CHX (10 μM) with or without PRQ (40 μM) for 0, 6, 12, and 24 h, and BCR-ABL protein levels were detected by immunoblotting. Folds of decrease of BCR-ABL protein/α-tubulin ratios were shown (*n* = 3). (N and O) K562R cells were exposed to PRQ (40 μM) in the presence or absence of MG132 (5 μM) for 24 h, and indicated protein levels were examined and quantitatively analyzed (*n* = 3). Data are presented as mean ± SD. One-way ANOVA; ns (not significant), ∗*p* < 0.05, ∗∗*p* < 0.01, and ∗∗∗*p* < 0.001 versus the control group.

An abnormality in the Wnt/β-catenin signaling pathway has been reported in CML cells. This pathway regulates the growth, differentiation, and self-renewal capacity of CML stem cells.^20^^,^^21^ To examine the impact of PRQ on Wnt signaling, we analyzed the level of the key mediator β-catenin. PRQ reduced β-catenin protein levels in a concentration-dependent manner (Figures 3A and 3B). As the stability of β-catenin protein is regulated by GSK-3beta activity, GSK-3beta activity was further examined. PRQ decreased the level of *p*-GSK-3β (Ser9)—a modification associated with GSK-3β inhibition—while increasing the levels of *p*-GSK-3β (Y216), which are linked to GSK-3β activation (Figures 3A–3D). To assess β-catenin transcriptional activity, we performed the TOP-flash luciferase reporter assay. PRQ significantly inhibited the β-catenin/TCF transcriptional activity (Figure 3E). These results demonstrated that PRQ inhibits the Wnt/β-catenin signaling pathway via enhancement of GSK-3beta activity. Importantly, CHIR-99021 co-treatment partially reversed PRQ-induced growth inhibition (Figure 3F). This partial rescue demonstrates that β-catenin downregulation functionally contributes to the anti-leukemic effects of PRQ.

To further explore the mechanism of PRQ in CML inhibition, the key oncogenic driver in CML, BCR-ABL, was analyzed. Intriguingly, PRQ resulted in a dose-dependent (Figures 3G and 3H) and time-dependent reduction (Figures 3I and 3J) of BCR-ABL protein levels. However, PRQ had no significant effect on *BCR::ABL1* transcript abundance in K562R cells (Figure 3K), indicating that PRQ affects the post-transcriptional process of BCR-ABL. To confirm this, K562R cells were treated with the protein synthesis inhibitor cycloheximide (CHX) with or without PRQ. PRQ accelerated the turnover of BCR-ABL protein, indicating that PRQ treatment reduced the protein stability of BCR-ABL (Figures 3L and 3M). The decrease of BCR-ABL protein was effectively abrogated in the presence of the proteasome inhibitor MG132 (Figures 3N and 3O), indicating the proteasome-dependent degradation of BCR-ABL protein.

Forced expression of BCR-ABL in 32D cells significantly increased β-catenin protein levels (Figures S4A and S4B), demonstrating that BCR-ABL could regulate β-catenin. In the CHIR-99021 rescue experiment, CHIR-99021 restored β-catenin levels but did not rescue BCR-ABL protein levels (Figures S4C and S4D), indicating that β-catenin downregulation does not feedback to regulate BCR-ABL expression.

Furthermore, PRQ did not induce HSP70 upregulation or disrupt HSP90-BCR-ABL interaction, suggesting that classical HSP90 inhibition is not the primary mechanism. Instead, PRQ promoted CRBN-BCR-ABL association, suggesting E3 ligase recruitment (Figures S5A–S5D).

### PRQ decreases the levels of clinically relevant BCR-ABL mutants

**Figure 4:**
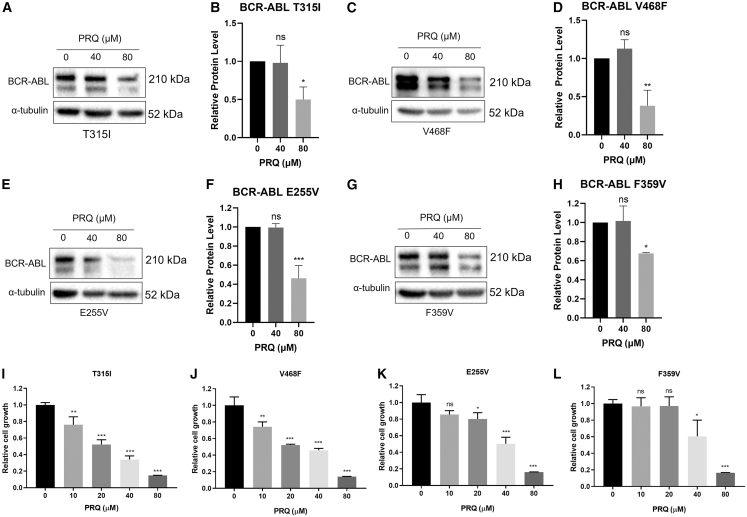
PRQ decreases the levels of clinically relevant BCR-ABL mutants (A–H) 32D cells expressing T315I (A and B), V468F (C and D), E255V (E and F), or F359V (G and H) treated with 0, 40, and 80 μM PRQ for 48 h were subjected to immunoblotting with the indicated antibodies and quantitatively analyzed (*n* = 3). (I–L) 32D cells expressing T315I (I), V468F (J), E255V (K), or F359V (L) were treated with 0, 10, 20, 40, and 80 μM PRQ for 48 h. The growth of cells was examined by CCK-8 assay (*n* = 3). Data are presented as mean ± SD. One-way ANOVA; ns (not significant), ∗*p* < 0.05, ∗∗*p* < 0.01, and ∗∗∗*p* < 0.001 versus control group.

Mutations in the *BCR::ABL1* gene lead to resistance to imatinib or other TKIs in patients with CML, representing a major clinical challenge.^22^ To investigate whether PRQ decreases mutant BCR-ABL protein levels, 32D cells stably expressing various BCR-ABL mutants were treated with PRQ. Parental 32D cells cultured without IL-3 underwent rapid cell death, confirming IL-3 dependence (Figure S6A). In contrast, 32D-BCR-ABL cells cultured without IL-3 proliferated normally (Figure S6B). PRQ significantly decreased the levels of BCR-ABL P210 T315I (Figures 4A and 4B), V468F (Figures 4C and 4D), E255V (Figures 4E and 4F), and F359V mutants (Figures 4G and 4H). Furthermore, PRQ exhibited suppression of proliferation in 32D cells expressing BCR-ABL mutants under IL-3-depleted conditions (Figures 4I–4L). Collectively, these findings demonstrate that PRQ displayed potential ability to treat CML cells harboring drug-resistant BCR-ABL mutants.

### PRQ induces differentiation and colony formation inhibition of CML primary cells

**Figure 5:**
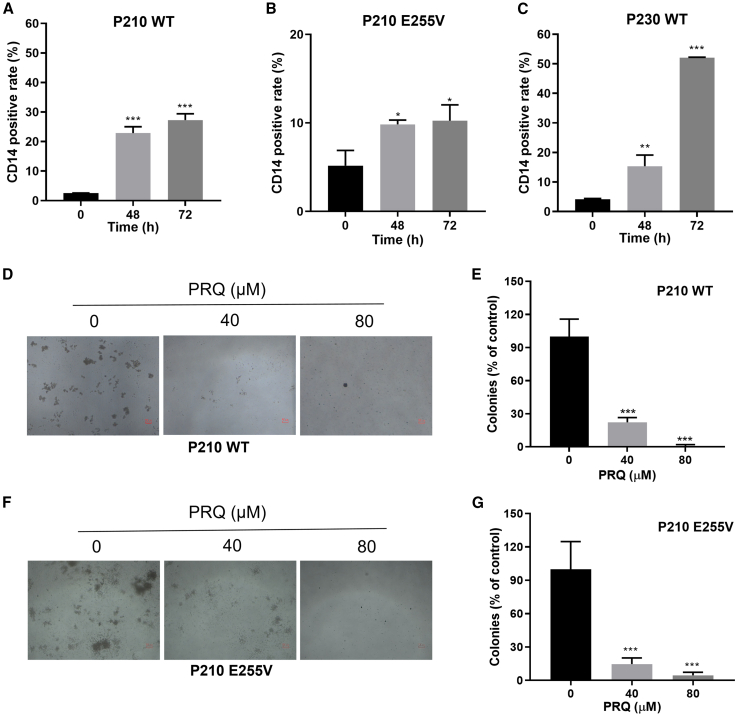
PRQ induces differentiation and colony formation inhibition of CML primary cells (A–C) Primary CML cells from 3 patients with CML were isolated and treated with 40 μM PRQ expressing BCR-ABL^210WT^ (A), BCR-ABL^210E255V^ (B), or BCR-ABL^230WT^ (C) for 0, 48, and 72 h, respectively, and the CD14 positive expression rate of the primary cells was examined by flow cytometry. (D and E) Primary CML cells expressing BCR-ABL^210WT^ were cultured in methylcellulose-based media containing PRQ (0, 40, and 80 μM). Colonies were quantified following two-week incubation, with representative photomicrographs to show morphological differences of colonies (40×). The values represent the inhibition % of colony formation compared with the control. (F and G) Primary CML cells expressing BCR-ABL^210E255V^ were cultured in methylcellulose-based media containing PRQ (0, 40, and 80 μM). Colonies were quantified following two-week incubation, with representative photomicrographs to show morphological differences of colonies (40×). The values represent the inhibition % of colony formation compared with the control. Data are presented as mean ± SD. One-way ANOVA, ∗*p* < 0.05, ∗∗*p* < 0.01, and ∗∗∗*p* < 0.001 versus the control group.

To investigate the effect of PRQ on the differentiation of CML primary cells, primary CML cells expressing BCR-ABL^210WT^, BCR-ABL^210E255V^, and BCR-ABL^230WT^ from 3 patients with CML were isolated and treated with PRQ. The results showed that PRQ increased CD14 expression in the primary cells expressing BCR-ABL^210WT^ (Figure 5A), BCR-ABL^210E255V^ (Figure 5B), and BCR-ABL^230WT^ (Figure 5C) from patients with CML. Next, we investigate whether PRQ has an inhibitory effect on the colony-forming ability of CML primary cells. PRQ treatment markedly reduced the clonogenic capacity of primary CML cells with BCR-ABL P210 wild-type (Figures 5D and 5E) and E255V mutant (Figures 5F and 5G).

### PRQ suppressed the neoplastic growth of imatinib-resistant CML cells in the xenograft model

**Figure 6:**
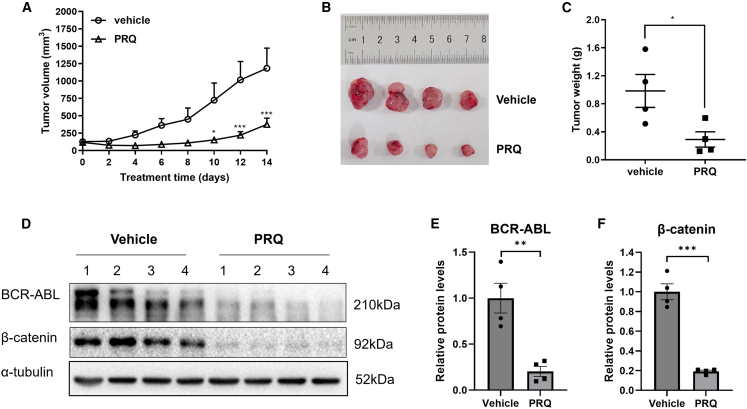
PRQ suppressed the neoplastic growth of imatinib-resistant CML cells in the xenograft model (A) K562R cells were subcutaneously injected into BALB/c nude mice. Ten days after cell implantation, mice were randomized and treated with vehicle or PRQ (40 mg/kg) by oral gavage for 14 days. Tumor dimensions were recorded at two-day intervals to calculate volume. (B) Photographic comparison of xenograft tumors excised from mice following a 14-day treatment regimen with either vehicle or PRQ. (C) The net tumor weight was determined following a 14-day treatment period with either vehicle or PRQ. (D) Lysates derived from xenograft tumors following treatment with vehicle or PRQ for 14 days were subjected to immunoblot analysis using the indicated antibodies. (E and F) The levels of indicated proteins were quantified. *n* = 4 mice for each group. Data are presented as mean ± SEM. Two-way RM ANOVA for (A), unpaired two-tailed *t* test for (C, E, F). ∗*p* < 0.05, ∗∗*p* < 0.01, and ∗∗∗*p* < 0.001 versus the control group.

To evaluate the *in vivo* antitumor efficacy of PRQ, a subcutaneous xenograft model was established using imatinib-resistant CML cells. Ten days after implantation, mice were randomized and orally given vehicle or PRQ for 14 days. The growth of K562R cells in mice was monitored. PRQ treatment significantly suppressed tumor growth in mice compared to vehicle-treated controls (Figure 6A). No significant body weight loss or overt signs of toxicity were observed in PRQ-treated mice (Figure S7). Treatment with PRQ resulted in a marked reduction in both mean tumor volume (Figure 6B) and tumor weight (Figure 6C) compared to the control group. Consistent with *in vitro* findings, PRQ treatment significantly decreased β-catenin and BCR-ABL protein levels in tumor xenografts (Figures 6D–6F).

## Discussion

Treatment with TKIs by targeting BCR-ABL has markedly improved clinical outcomes in patients with *BCR::ABL1*^+^ CML. Imatinib, the first generation of TKI, serves as a first-line drug for the treatment of CML. However, drug resistance remains a critical therapeutic challenge in the management of CML.^23^ In our previous study, we demonstrated that PRQ, which is widely used to treat or prevent relapse of malaria, exhibited potent anti-leukemic effects in acute myeloid leukemia models.^16^ This investigation reveals that PRQ effectively suppresses the proliferation of imatinib-resistant CML cells through the induction of cellular differentiation and apoptosis. Moreover, PRQ induces differentiation and colony formation inhibition of CML primary blasts, and significantly suppresses the neoplastic growth of imatinib-resistant CML cells in a murine xenograft model. These preclinical findings collectively suggest the therapeutic potential of PRQ for overcoming TKI resistance in patients with CML.

Our results demonstrated that PRQ exhibited suppression on the proliferation of imatinib-resistant CML cells with minimal cytotoxic effects on normal neutrophils and PBMCs at equivalent concentrations. Notably, PRQ significantly inhibited the clonogenic growth of CML cells in a dose-dependent manner (Figure 1). It is noteworthy that lower concentrations of PRQ induced monocytic differentiation of K562R cells, whereas higher concentrations of PRQ induced apoptosis in K562R cells (Figure 2). These dual pharmacological effects suggest that PRQ exerts its anti-proliferative activity through distinct molecular pathways depending on its concentrations.

Our mechanistic studies support a model that PRQ induces differentiation and apoptosis in imatinib-resistant CML cells through dual mechanisms: (1) inhibition of the Wnt/β-catenin pathway and (2) acceleration of BCR-ABL protein degradation (Figure 3).

The Wnt/β-catenin pathway, known to drive CML progression by sustaining leukemic stem cell self-renewal, is critically regulated by GSK-3β kinase activity.^20^^,^^21^^,^^24^ The stimulation of Wnt signaling leads to a decrease in GSK-3beta activity, resulting in the nuclear accumulation of β-catenin and transactivation of transformed cell genes.^25^ Phosphorylation of GSK-3β at serine 9 reduces the activity of GSK-3β kinase, whereas phosphorylation of GSK-3β at tyrosine 216 and GSK-3α at tyrosine 279 increase the kinase activity of these two isoforms.^26^ Our data showed that PRQ increased GSK-3beta activity, decreased β-catenin protein level, and inhibited the β-catenin/TCF transcriptional activity. These findings demonstrate that PRQ suppresses the Wnt/β-catenin signaling pathway. Restoration of β-catenin levels partially reversed PRQ-induced growth suppression, demonstrating that β-catenin downregulation functionally contributes to the anti-leukemic activity. The partial rescue reflects the multi-targeted nature of PRQ, which also degrades oncogenic protein BCR-ABL.

PRQ-induced BCR-ABL degradation is proteasome-dependent, as evidenced by MG132 blockade. While HSP90 is known to maintain BCR-ABL stability, we did not detect HSP70 upregulation or a constitutive HSP90-BCR-ABL interaction, suggesting that classical HSP90 inhibition is not the primary mechanism. PRQ treatment increased CRBN-BCR-ABL interaction, suggesting recruitment of this E3 ligase. PRQ has been reported to affect redox balance. A potential contribution to BCR-ABL destabilization cannot be excluded and warrants future investigation.

PRQ upregulated PU.1 at both the mRNA and protein levels, consistent with transcriptional derepression upon BCR-ABL degradation via the MYC/miR-150/MYB/miR-155 network.^27^ In contrast, C/EBPα was upregulated only at the protein level, consistent with translational derepression upon BCR-ABL inhibition via hnRNP E2.^28^ Thus, the mechanistic data suggest a model: PRQ-induced BCR-ABL degradation relieves the differentiation block through dual mechanisms—transcriptional activation of PU.1 and translational activation of C/EBPα—converging to induce monocytic differentiation.

Our data also revealed that PRQ induces the dose-dependent degradation of BCR-ABL fusion protein with the concomitant reduction of mutant BCR-ABL variants (T315I, E255V, F359V, and V468F) that confer resistance to different TKIs (Figure 4). Of these mutations, T315I is the most prevalent gatekeeper mutation and confers broad resistance to available ATP-competitive TKIs except ponatinib, whose clinical utility is constrained by thrombotic risks.^29^^,^^30^ E255V is also a gatekeeper mutation that confers robust resistance to imatinib while exhibiting intermediate resistance to both nilotinib and ponatinib.^31^ The F359V and V468F mutations are established as the primary mechanisms mediating resistance to asciminib, an STAMP inhibitor targeting the ABL myristoyl pocket.^32^^,^^33^ Our results demonstrated that PRQ has efficacy on both primary and secondary imatinib-resistant CML.

Our work demonstrates that PRQ induces differentiation and colony formation inhibition of CML primary blasts expressing BCR-ABL wild type or mutant proteins (Figure 5). Furthermore, PRQ suppresses the neoplastic growth of imatinib-resistant CML cells in a murine xenograft model (Figure 6).

In summary, our study establishes the inhibitory efficacy of PRQ against imatinib-resistant CML cells both *in vitro* and *in vivo*. This anti-leukemic effect is mediated through dual mechanisms: inhibition of the Wnt/β-catenin signaling pathway and acceleration of BCR-ABL protein degradation. These findings provide a rationale for further investigation of PRQ as a potential therapeutic agent for imatinib-resistant CML.

### Limitations of the study

Several limitations of this study should be acknowledged. The molecular mechanism by which PRQ induces BCR-ABL proteolysis remains to be fully elucidated. The primary CML patient samples were limited, precluding definitive conclusions about efficacy in specific subpopulations. The *in vivo* experiment used a small cohort and should be considered a pilot study. Additionally, the PRQ concentrations used in this study exceed clinically achievable plasma levels, and off-target effects cannot be completely excluded. Nevertheless, our findings provide a mechanistic proof-of-concept that PRQ targets BCR-ABL and Wnt/β-catenin pathways, warranting further medicinal chemistry optimization and validation in larger cohorts.

## Resource availability

### Lead contact

Requests for further information and resources should be directed to and will be fulfilled by the lead contact, Licai He (helicai@wmu.edu.cn).

### Materials availability

This study did not generate new unique reagents.

### Data and code availability

• Data reported in this paper will be shared by the lead contact upon request.
• This article does not report original code.
• Any additional information required to reanalyze the data reported in this article is available from the lead contact upon request.

## Acknowledgments

We would like to thank Dr. Qianqian Yin from ShanghaiTech University for generously providing the 32D cells that express different imatinib-resistant BCR-ABL mutants. This research was supported by the 10.13039/501100004731Zhejiang Provincial Natural Science Foundation of China under grant no. LTGY23H080005, National Natural Science Foundation of China (82272702, 82302925), Wenzhou Science and Technology Bureau of China (Y20240037), and in part supported by the 10.13039/501100017528Key Discipline of Zhejiang Province in Medical Technology (First Class, Category A).

## Author contributions

L.H. and H.G. designed the research. C.Y. and W.L. performed most experiments and analyzed the data. C.M. and Z.Z. performed the cell growth and colony-forming assay. J.C. and G.W. wrote animal protocols. C.Y. and Z.F. performed the experiment using primary human CML samples. L.H. and H.G. wrote the manuscript. All authors reviewed and approved the manuscript.

## Declaration of interests

The authors have filed a patent application related to this work (application pending), in which authors L.H., C.Y., H.G., and W.L are listed as co-inventors. The remaining authors declare no competing interests.

## STAR★Methods

### Key resources table

**Table:** 

REAGENT or RESOURCEkey-resources-table	SOURCE	IDENTIFIER
**Antibodies**		

α-tubulin	Cell Signaling Technology	Cat#3873
C/EBP-α	Cell Signaling Technology	Cat#8178
PU.1	Cell Signaling Technology	Cat#2258
Cleaved Caspase-3	Cell Signaling Technology	Cat#9661
Cleaved PARP	Cell Signaling Technology	Cat#5625
*p*-GSK-3β (Ser9)	Cell Signaling Technology	Cat#5558
beta-Catenin	Cell Signaling Technology	Cat#8480
c-Abl	Cell Signaling Technology	Cat#2862
CRBN	Cell Signaling Technology	Cat#71810
*p*-GSK3β (Y216)+p-GSK3α (Y279)	Abcam	Cat#ab68476
HSP70	Beyotime Biotechnology	Cat#AF0189
HSP90	Santa Cruz Biotechnology	Cat#sc-13119
FITC anti-human CD14	BioLegend	Cat#301803
FITC anti-human CD15 (SSEA-1)	BioLegend	Cat#323003
FITC anti-human CD235a (Glycophorin A)	BioLegend	Cat#349103
HRP-labeled Goat Anti-Rabbit IgG(H + L)	Beyotime Biotechnology	Cat#A0352
HRP-labeled Goat Anti-Mouse IgG(H + L)	Beyotime Biotechnology	Cat#A0216

**Bacterial and virus strains**		

DH5α Competent cell	Vazyme	Cat#C502

**Biological samples**		

Human blood samples	Jinhua Central Hospital	N/A

**Chemicals, peptides, and recombinant proteins**		

Imatinib	Selleckchem	Cat#S2475
Primaquine diphosphate (PRQ)	MedChemExpress	Cat#HY-12651
CHIR-99021	Selleckchem	Cat#S2924
Cycloheximide (CHX)	Selleckchem	Cat#S7418
MG132	Selleckchem	Cat#S2619
NBT	Beyotime Biotechnology	Cat#ST362
TPA	Beyotime Biotechnology	Cat#S1819

**Critical commercial assays**		

Cell Counting Kit-8	Dojindo Laboratories	Cat#CK04
Luc-Pair™ Duo-Luciferase HS Assay Kit	GeneCopoeia	Cat#LF004
Annexin V-PE/7-AAD apoptosis kit	Vazyme	Cat#A213
Matrigel	Corning	Cat#354234
MethoCult H4434 Classic	STEMCELL Technologies	Cat#04434CN
Human peripheral blood neutrophil separation medium kit	tbdscience (TBD)	Cat#LZS11131
Human peripheral blood mononuclear cell isolation kit	tbdscience (TBD)	Cat#LDS1075
Taq Pro Universal SYBR qPCR Master Mix	Vazyme	Cat#Q712

**Experimental models: Cell lines**		

293 T/17	American type culture collection	CRL-11268
K562	American type culture collection	CCL-243
32D cells expressing BCR-ABL mutants	ShanghaiTech University.	N/A

**Experimental models: Organisms/strains**		

BALB/c female nude mice (6-8-week-old)	GemPharmatech	N/A

**Oligonucleotides**		

BCR::ABL1-F:TCCACAGCATTCCGCTGAC	GENEWIZ, Suzhou, China	N/A
BCR::ABL1-R:TTTGAGCCTCAGGGTCTGAGTG	GENEWIZ, Suzhou, China	N/A
GAPDH-F:CAGGAGGCATTGCTGATGAT	GENEWIZ, Suzhou, China	N/A
GAPDH-R:GAAGGCTGGGGCTCATTT	GENEWIZ, Suzhou, China	N/A
CEBPA-F:AGGAGGATGAAGCCAAGCAGCT	GENEWIZ, Suzhou, China	N/A
CEBPA-R:AGTGCGCGATCTGGAACTGCAG	GENEWIZ, Suzhou, China	N/A
SPI1-F:GACACGGATCTATACCAACGCC	GENEWIZ, Suzhou, China	N/A
SPI1-R:CCGTGAAGTTGTTCTCGGCGAA	GENEWIZ, Suzhou, China	N/A

**Software and algorithms**		

FlowJo	FlowJo software	N/A
GraphPad Prism 7.0	GraphPad Prism 7.0 software	N/A
ImageLab	ImageLab software	N/A
SnapGene	SnapGene software	N/A
ImageJ	ImageJ software	N/A

### Experimental model and study participant details

#### Cell lines

The female human embryonic kidney cell line 293T/17 and the female human chronic myelogenous leukemia cell line K562 were purchased from American Type Culture Collection (ATCC), which were authenticated by the vendors using short tandem repeat (STR) profiling prior to shipment. Imatinib-resistant K562 cells (K562R) were established through gradual dose escalation of imatinib from 0.1 μM to 3 μM over three months. The IC_50_ of imatinib for K562R cells was 10.59-fold higher than that for parental K562 cells. K562R cells were maintained in the presence of 1 μM imatinib to preserve the resistant phenotype. The male murine hematopoietic 32D cells expressing different imatinib resistant BCR-ABL mutants were generously provided by Dr. Qianqian Yin of ShanghaiTech University. The generation and characterization of these mutant cell lines have been described previously.^34^

All cell lines were confirmed negative for mycoplasma contamination. 293T/17 were maintained in DMEM medium (Gibco BRL, Gaithersburg, MD) supplemented with 10% fetal bovine serum (FBS). K562, K562R and 32D cell lines were maintained in RPMI-1640 medium (Gibco BRL, Gaithersburg, MD) supplemented with 10% FBS. All cell lines were maintained at 37°C under a humidified atmosphere of 5% CO_2_.

#### Xenograft model

BALB/c (*Foxn1*^nu^/*Foxn1*^nu^) female nude mice (6-8-week-old) from GemPharmatech (Nanjing, China) were housed under specific pathogen-free (SPF) conditions with free access to food and water. Mice were treated with cyclophosphamide (100 mg/kg/day) for 3 consecutive days prior to inoculation. K562R cells (2×10^6^) mixed with Matrigel (Corning, NY, USA) were subcutaneously transplanted into mice. Ten days after implantation, mice were randomly divided into two groups. PRQ was dissolved in sterile water and administered by oral gavage at a dose of 40 mg/kg once daily for 14 consecutive days. The control group received an equal volume of vehicle (sterile water). Tumor size was monitored every two days. Tumor volume was calculated by the following formula: V= (long diameter) × (short diameter)^2^ × 1/2. All experimental procedures involving animals were conducted in compliance with the ARRIVE guidelines and approved by the Institutional Animal Care and Use Committee of Wenzhou Medical University (wydw2022-0905).

#### Human samples

Peripheral blood samples were obtained from three male patients with CML (aged 34-51 years, Table S1) at Affiliated Jinhua Hospital, Zhejiang University School of Medicine. The patients were of East Asian (Chinese) ancestry. All participants provided written informed consent, and the study protocol was approved by the Institutional Review Board at Jinhua Hospital (2025-295).

#### Sex as a biological variable

The influence of sex as a biological variable was not analyzed, as no single experimental model included both sexes. We acknowledge this limitation.

### Method details

#### Chemical reagents and compounds

PRQ was purchased from MedChem Express (MCE, USA). Imatinib, Cycloheximide (CHX), MG132 and CHIR99021 were purchased from Selleckchem (Houston, TX, USA).

#### Cell growth and viability assays

Cells were seeded in 96-well plates at 2×10^5^ cells/mL, and treated with various dilutions of the compounds for 48 hours. Cell proliferation was assessed using the CCK-8 assay (Dojindo Laboratories, Kumamoto, Japan), and cell viability was assessed by trypan blue exclusion, respectively, following the manufacturers' protocols.

#### NBT reduction assay

NBT reduction assay was used to determine the differentiation of K562 and K562R cells into myeloid cells. Cells were seeded in 24-well plates at 2×10^5^ cells/mL, and treated with 0, 20 and 40 μM PRQ for 2 days. After teatment, cells were incubated with 0.2% nitroblue tetrazolium (NBT) solution containing 100μg/ml TPA (Beyotime Biotechnology, Shanghai, China) for 30 min at 37°C. Subsequently, cells were collected onto slides by cytospin, fixed with formaldehyde and stained with Wright-Giemsa dye. A minimum of 200 cells were counted to obtain the percentage of positive cells.

#### Flow cytometry

K562R cells were seeded in 6-well plates at 2×10^5^ cells/mL and treated with PRQ (0, 20 and 40 μM) for 48 h. Cells were then stained with FITC anti-human CD14, FITC anti-human CD235a, or FITC anti-human CD15 antibodies (Biolegend, USA). For each sample, debris was excluded by gating on FSC-A vs. SSC-A, and doublets were excluded by gating on FSC-A vs. FSC-H. Positive populations for each marker were determined using respective isotype controls.

For apoptosis analysis, K562R cells were seeded in 6-well plates at 2×10^5^ cells/mL, and treated with different concentrations of PRQ (0, 40 and 80 μM) for 48 h. Annexin V-PE/7-AAD apoptosis detection kit (Vazyme Biotech, Nanjing, China) was used to examine cell apoptosis according to the manufacturer’s protocol.

Flow cytometry was used to detect the CD14 expression and apoptotic cells. FlowJo software was used to analyze flow cytometry data.

#### Western blot analysis

Cells were seeded at 2×10^5^ cells/mL, and treated under conditions specified in each figure legend. Cell protein extracts were separated by SDS-PAGE electrophoresis and then transferred to polyvinylidene fluoride (PVDF) membranes for detection as previously described.^35^ Following transfer, the membranes were subjected to blocking with 5% non-fat milk and subsequently probed with primary antibodies. After washing, membranes were then probed with horseradish peroxidase (HRP)-conjugated secondary antibodies (Beyotime Biotechnology, Shanghai, China). Protein bands were visualized using an Enhanced Chemiluminescence (ECL) substrate and imaged with a ChemiDoc MP system (Bio-Rad).

#### Immunoprecipitation (IP)

K562R cells (2×10^5^ cells/mL) treated with vehicle or 40 μM PRQ for 24 h were collected and lysed in IP lysis buffer (50 mM Tris-HCl, pH 7.4, 150 mM NaCl, 1% Triton X-100, 20 mM β-glycerophosphate) supplemented with 10 mM NaF, 2 mM Na_3_VO_4_, and protease inhibitor cocktail (Bimake, Houston, TX, USA). The lysates were centrifuged to remove insoluble debris. The clarified supernatants were incubated with the appropriate primary antibodies overnight at 4°C. Antibody-bound proteins were captured using protein A agarose beads (REPLIGEN, Boston, MA, USA), washed with lysis buffer, and eluted by 1× SDS sample loading buffer. The eluted proteins were then analyzed by Western blotting.

#### Quantitative real-time PCR

Cells were seeded at 2×10^5^ cells/mL, and treated under conditions specified in each figure legend. Total RNA was extracted from cultured cells utilizing TRIzol reagent, following the manufacturer's protocol. Quantitative real-time PCR (qPCR) was subsequently performed in accordance with previously established methods.^16^ The specific primers employed in this assay were detailed in Table S2.

#### Isolation of primary CML cells

Primary CML cells were isolated from peripheral blood using a Human peripheral blood mononuclear cell isolation kit (TBDScience, Tianjin, China), according to the manufacturer’s instructions.

#### Colony forming assay

K562R cells (1000 cells per well) or primary cells from CML patients were mixed with methylcellulose medium H4434 (StemCell Technologies, Vancouver, BC, Canada), plated into 24-well plates, and treated with 0, 40 and 80 μM PRQ. After 2 weeks of incubation, colony-forming unit-granulocyte and macrophage (CFU-GM) were enumerated.

#### Luciferase assay

293T/17 cells were co-transfected with TOP flash-luciferase (firefly) reporter and TK-renilla luciferase plasmids. After transfection, cells were exposed to increasing concentrations of PRQ (0, 20, 40, and 80 μM) for 48 h. Luciferase activity was then measured using the Luc-Pair™ Luciferase Assay Kit (GeneCopoeia, USA) according to the manufacturer's protocols.

### Quantification and statistical analysis

Data analyses were performed using GraphPad Prism 7.0 software.Quantitative data from cell line experiments were derived from at least three independent biological replicates and are presented as mean ± SD or ± SEM. The unpaired two-tailed Student's t-test was utilized for two-group comparisons. One-way analysis of variance (ANOVA) was employed for multi-group comparisons. Two-way RM ANOVA was used for the analysis of tumor growth curves. Significance levels are denoted as: ∗p < 0.05, ∗∗p < 0.01, ∗∗∗p < 0.001.
