# Supplementary material for: Repurposing primaquine diphosphate for imatinib-resistant chronic myeloid leukemia via targeting BCR-ABL and Wnt/β-catenin pathway
Source: iScience. 2026 Jun 30;29(7):116415. doi: 10.1016/j.isci.2026.116415 (PMC13330541; doi:10.1016/j.isci.2026.116415)

iScience, Volume *29*

Supplemental information

Repurposing primaquine diphosphate for imatinib-resistant chronic myeloid leukemia via targeting BCR-ABL and Wnt/β-catenin pathway

Changqing Yin, Wenhao Liu, Chensheng Ma, Zhida Zhang, Zenghui Fang, Jiawei Cao, Guang Wu, Haihua Gu, and Licai He

Supplementary Information

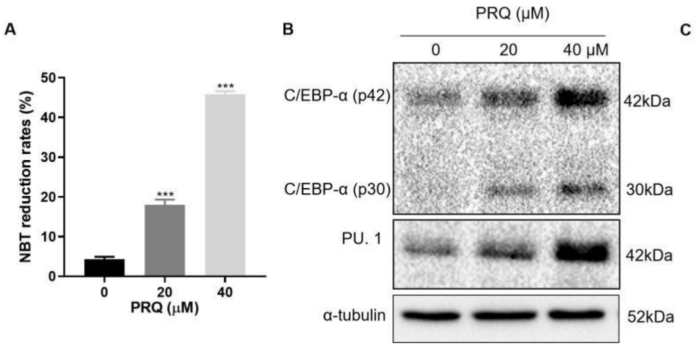

Figure S1. PRQ induces differentiation of K562 cells. (A) K562 cells were treated with 0, 20 and 40 μM PRQ for 48 h, and the rates of NBT reduction were shown. (B-C) K562 cells treated with 0, 20 and 40 μM PRQ for 48 h were immunoblotted with the indicated antibodies and quantitatively analyzed. Data are presented as mean ± SD. One-way ANOVA; ns (not significant),*P < 0.05, **P < 0.01, ***P <0.001 versus control group.

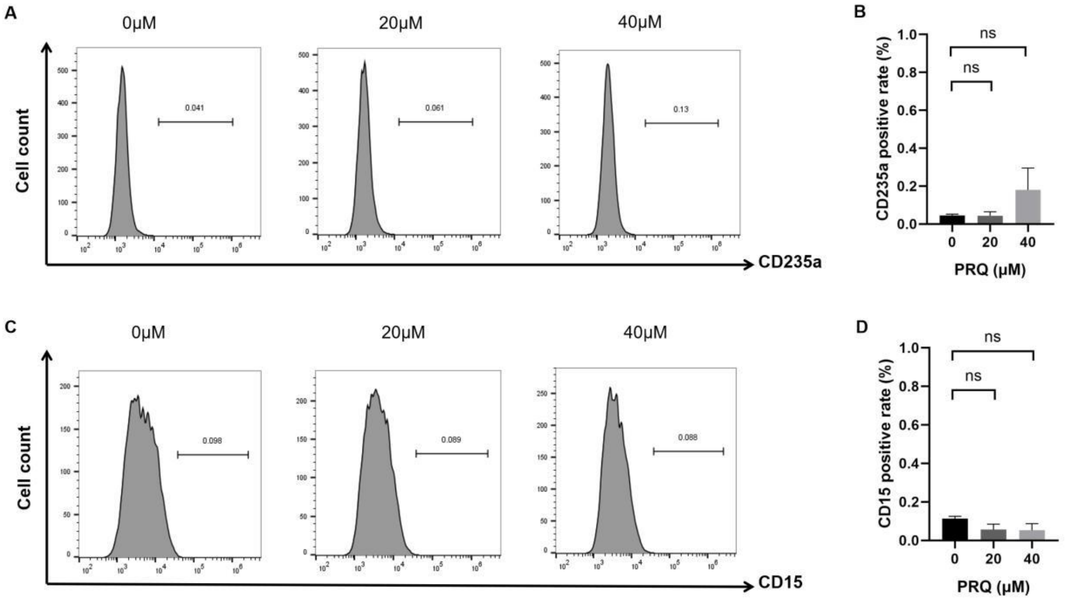

Figure S2. PRQ functions without affecting erythroid or granulocytic lineages.

(A-B) K562R cells were treated with 0, 20 and 40 μM PRQ for 48 h. The percentage of CD235a$^+$ cells were detected by flow cytometry. (C-D) K562R cells were treated with 0, 20 and 40 μM PRQ for 48 h. The percentage of CD15$^+$ cells were detected by flow cytometry. Data are presented as mean ± SD. One-way ANOVA; ns (not significant).

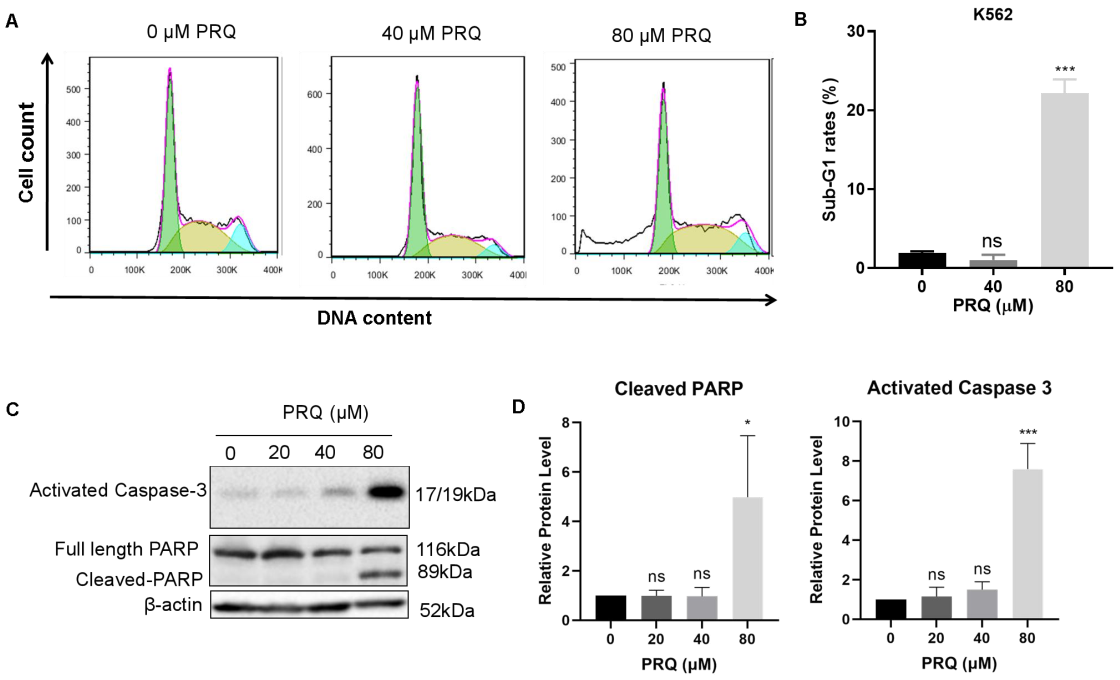

Figure S3. PRQ induces apoptosis of K562 cells. (A-B) K562 cells were treated with with 0, 40 and 80μM PRQ for 48 h, and flow cytometry was used to detect the percentage of sub-G1 cells after PI staining assay. (C-D) K562 cells treated with 0, 20, 40 and 80μM PRQ for 48 h were immunoblotted with the indicated antibodies and quantitatively analyzed. Data are presented as mean ± SD. One-way ANOVA; ns (not significant), *P < 0.05, ***P <0.001 versus control group.

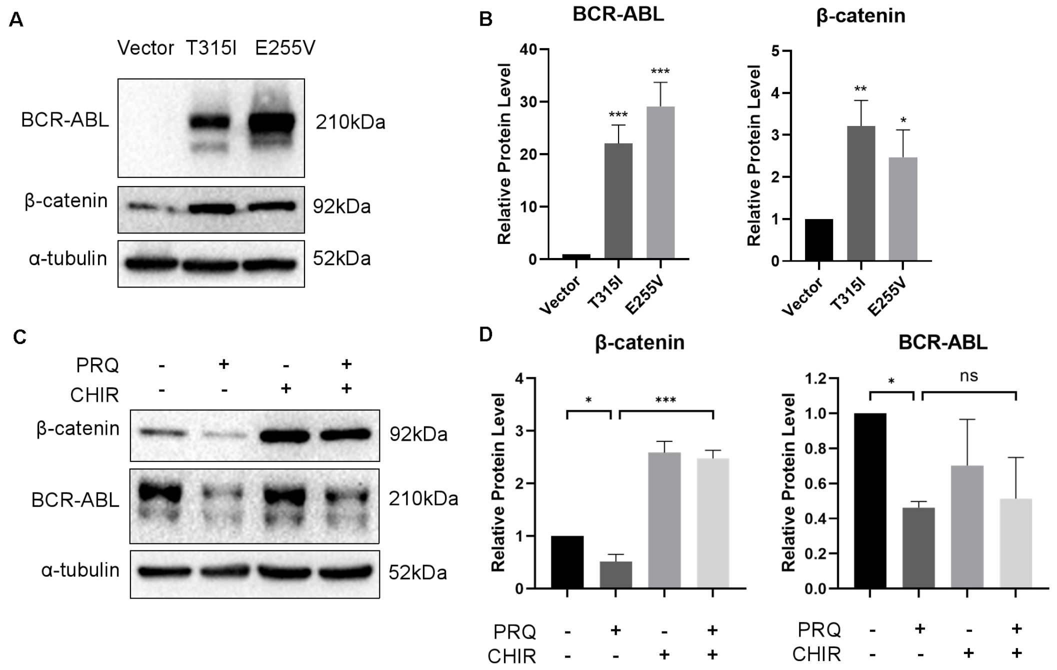

Figure S4. BCR-ABL regulates β-catenin. (A-B) 32D cells expressing Vector, T315I or E255V treated with 40 μM PRQ for 48 h were subjected to immunoblotting with the indicated antibodies and quantitatively analyzed. (C-D) K562R cells were treated with or without CHIR (1μM) and/or PRQ (40 μM) for 48 h. Cell proteins were subjected to immunoblotting with the indicated antibodies and quantitatively analyzed. Data are presented as mean ± SD. One-way ANOVA; ns (not significant), $^{*}P < 0.05$, $^{**}P < 0.01$, $^{***}P < 0.001$ versus control group.

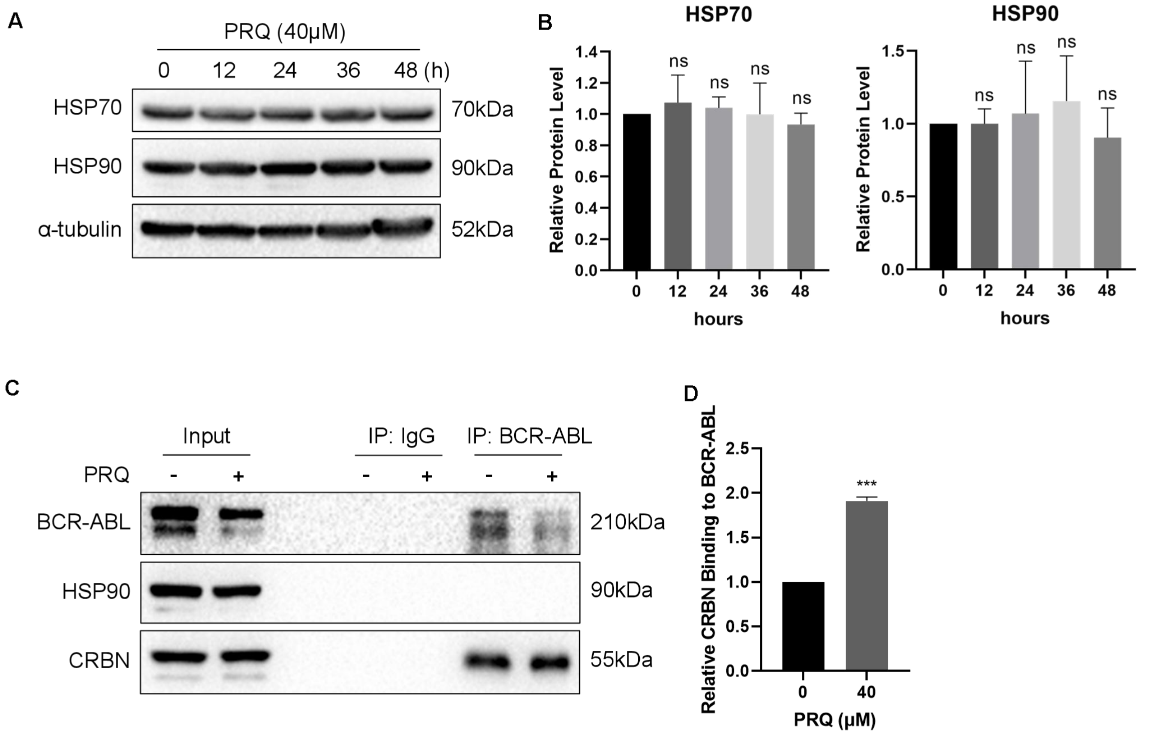

Figure S5. PRQ promotes CRBN recruitment. (A-B) K562R cells treated with 40 μM PRQ for 0, 12, 24, 36 and 48 h were subjected to immunoblotting with the indicated antibodies and quantitatively analyzed. (C-D) K562R cells were treated with vehicle or 40 μM PRQ for 24 h, and subjected to immunoprecipitation with anti-BCR-ABL antibody, and followed by immunoblotting with indicated antibodies. Relative CRBN binding to BCR-ABL was quantitatively analyzed. Data are presented as mean ± SD. One-way ANOVA for (B); unpaired two-tailed t-test for (D). ns (not significant), ***P <0.001 versus control group.

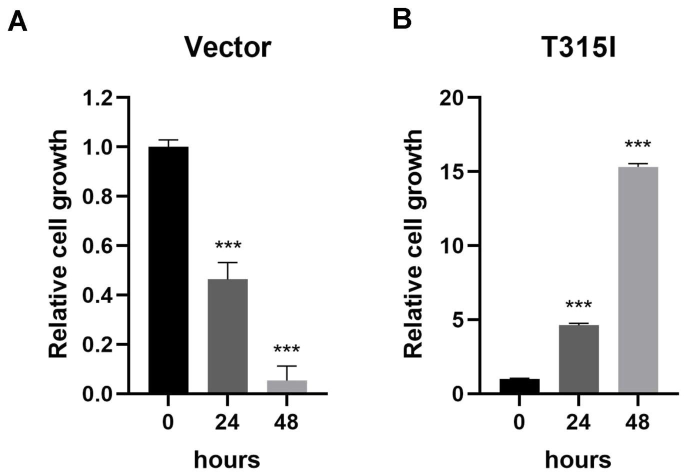

Figure S6. BCR-ABL expression confers IL-3-independent survival on 32D cells. (A-B) 32D cells expressing Vector or T315I were cultured in the medium without IL-3, and the growth of cells was examined using the CCK-8 assay. Data are presented as mean ± SD. One-way ANOVA; ***P <0.001 versus control group.

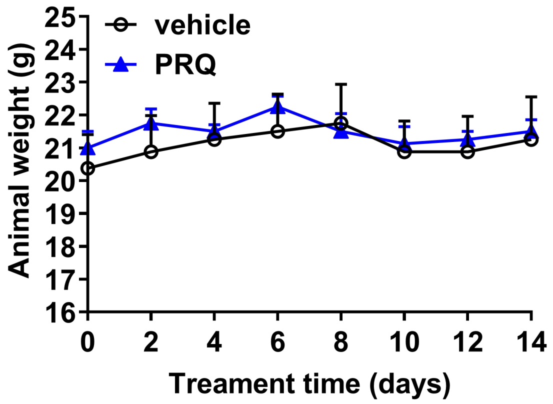

Figure S7. Body weight changes during PRQ treatment in a xenograft mouse model. Mice bearing K562R xenografts were treated with vehicle (sterile water) or PRQ (40 mg/kg, oral gavage, once daily) for 14 consecutive days. Body weights were measured every two days. Data are presented as mean ± SEM (n = 4). Two-way RM ANOVA revealed no significant differences between the two groups at any time point.

Table S1. Clinical characteristics of CML patients

Patient	Age (years)	Sex	Disease status	BCR-ABL transcript	Mutation status
#1	51	Male	CML-CP	P210	WT
#2	34	Male	CML-MBC	P210	E255V
#3	38	Male	CML-LBC	P230	WT

Abbreviations: CML-CP, chronic myeloid leukemia in chronic phase; CML-MBC, CML in myeloid blast crisis; CML-LBC, CML in lymphoid blast crisis; WT, Wild-type.

Table S2. The Primer sequence

Number	Primer name	Sequence (5’-3’)
1	BCR::ABL1-F	TCC ACA GCA TTC CGC TGA C
2	BCR::ABL1-R	TTT GAG CCT CAG GGT CTG AGT G
3	CEBPA-F	AGG AGG ATG AAG CCA AGC AGC T
4	CEBPA-R	AGT GCG CGA TCT GGA ACT GCA G
5	SPI1-F	GAC ACG GAT CTA TAC CAA CGC C
6	SPI1-R	CCG TGA AGT TGT TCT CGG CGA A
7	GAPDH-F	CAG GAG GCA TTG CTG ATG AT
8	GAPDH-R	GAA GGC TGG GGC TCA TTT

Data S1. Original western blot images for all figures

Figure 2H

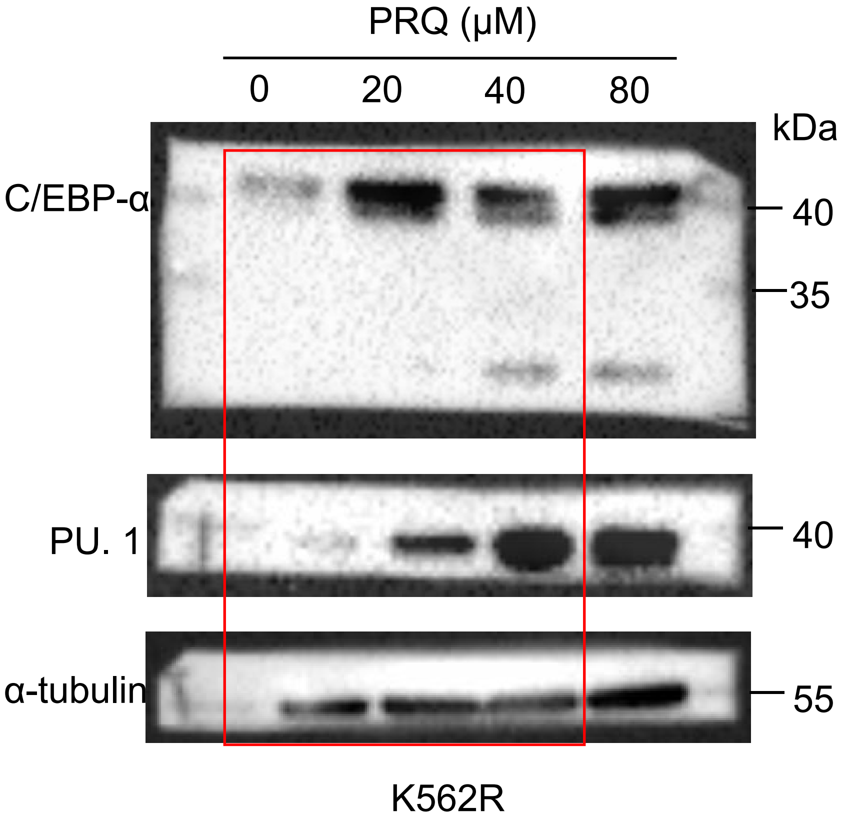

Figure 2L

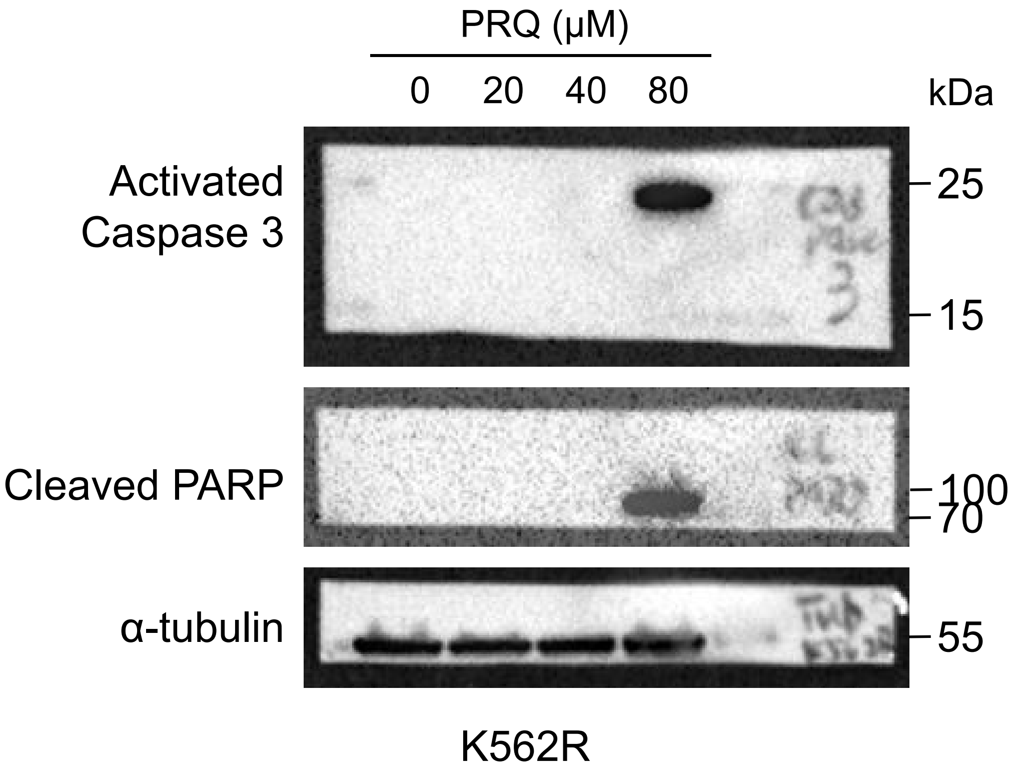

Figure 3A

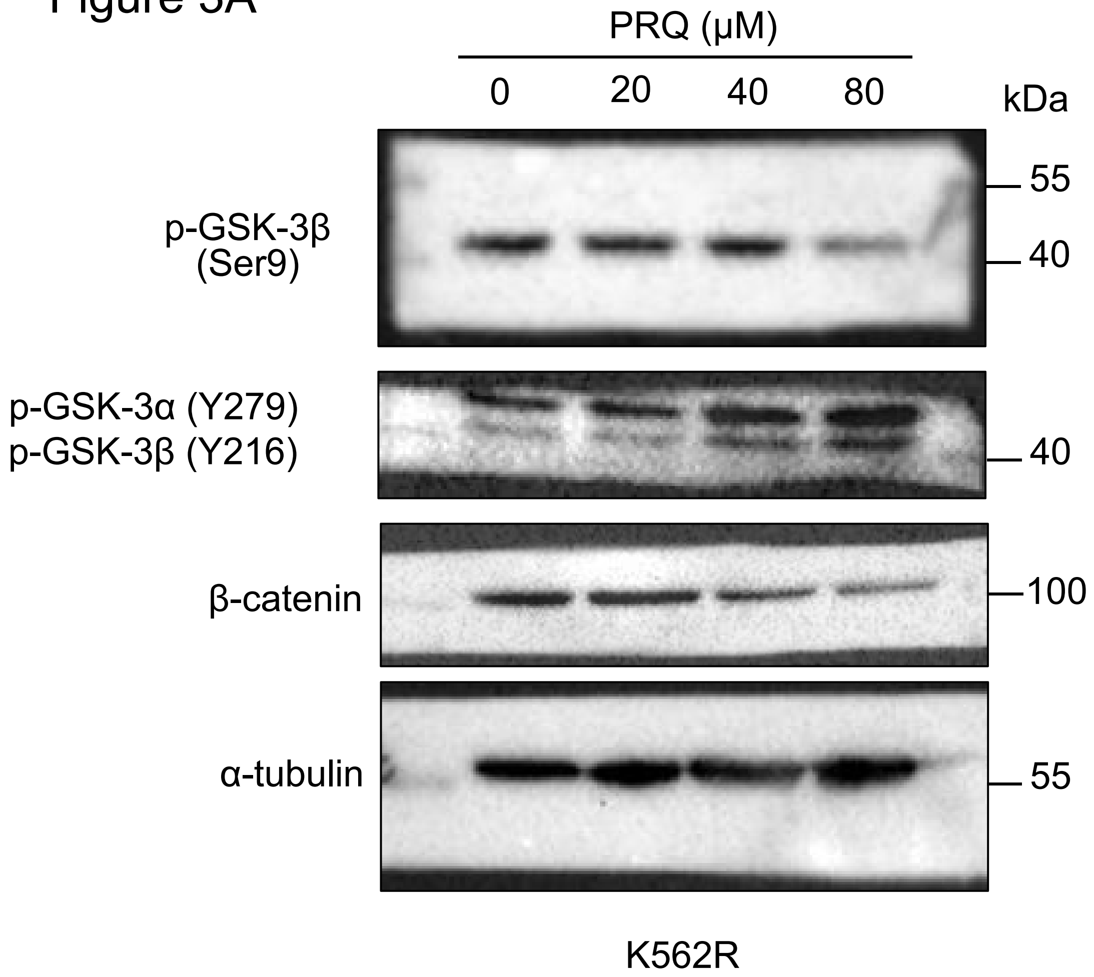

Figure 3C

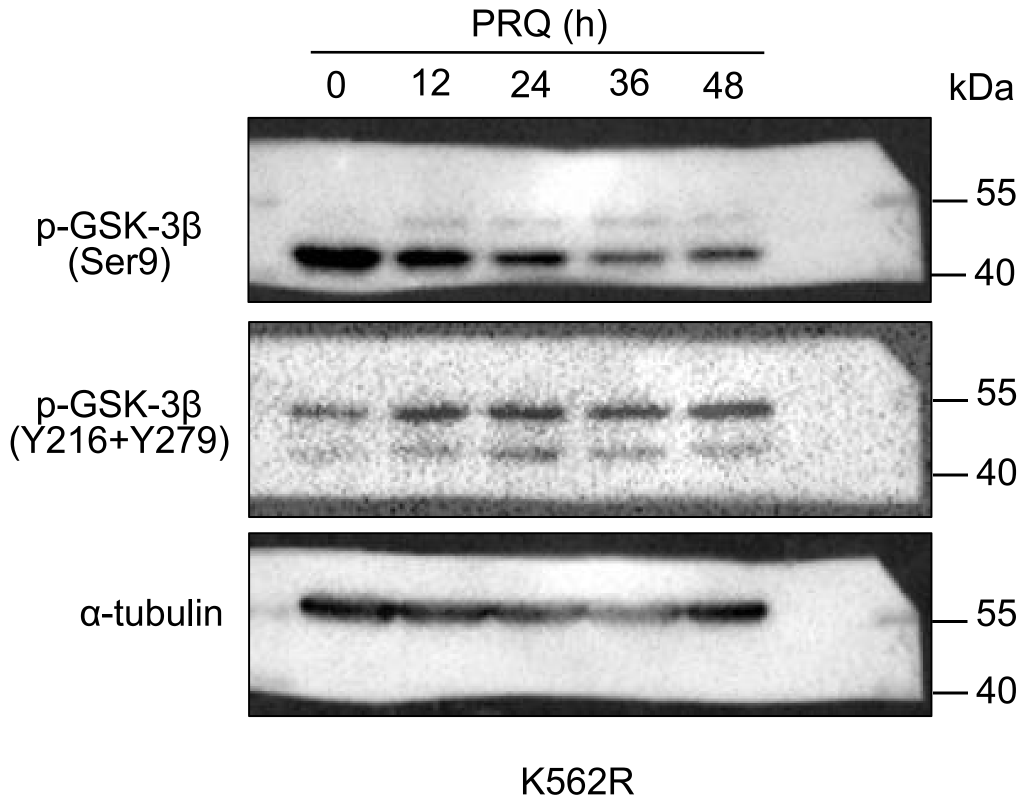

Figure 3G

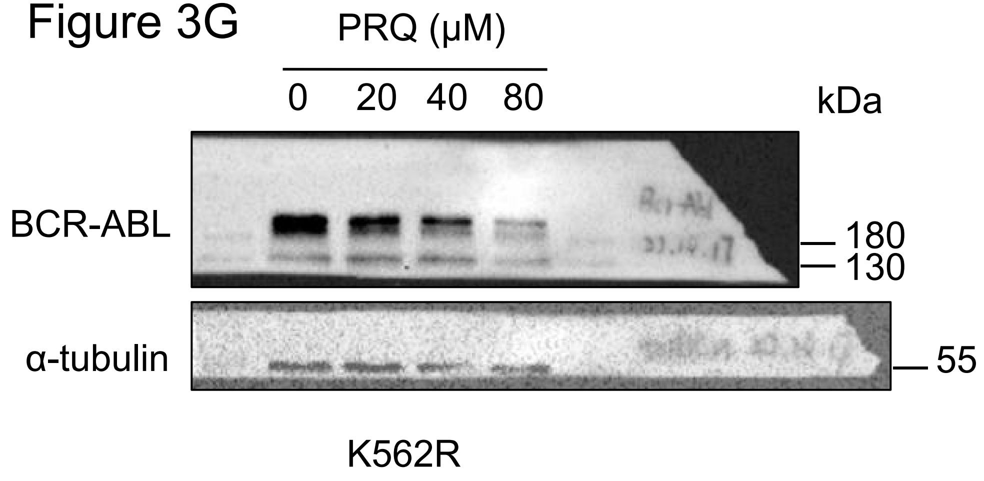

Figure 3L

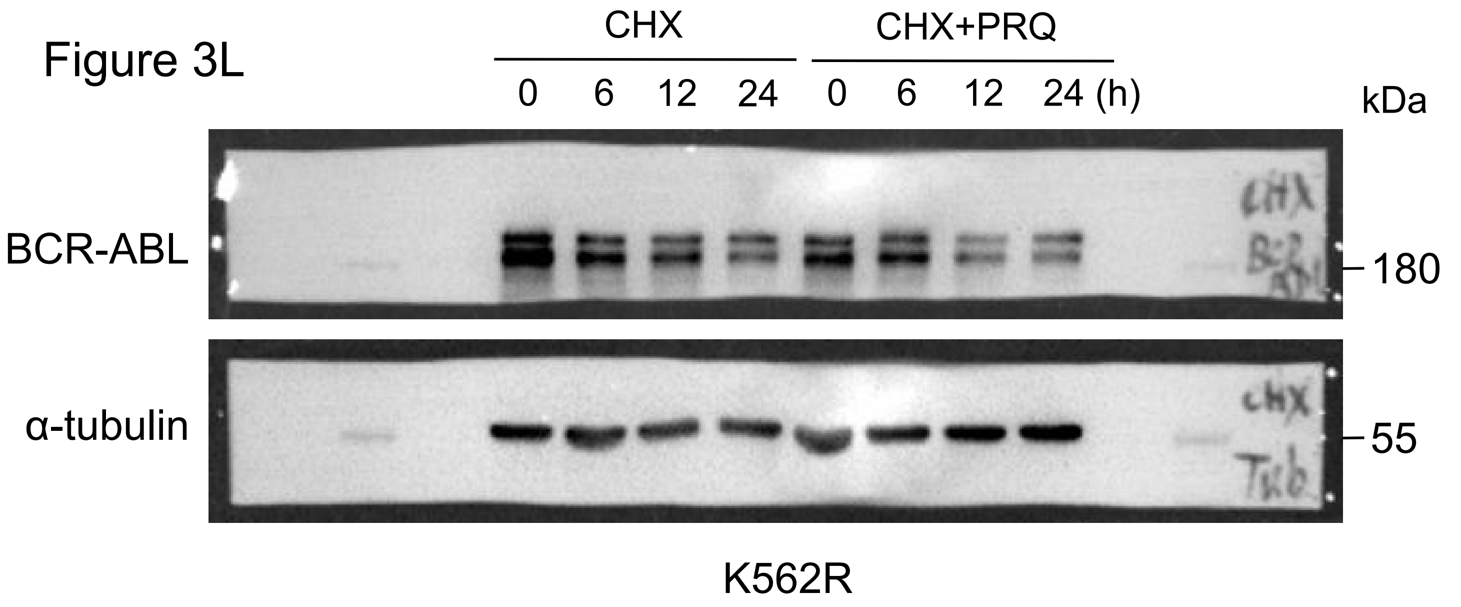

Figure 3I

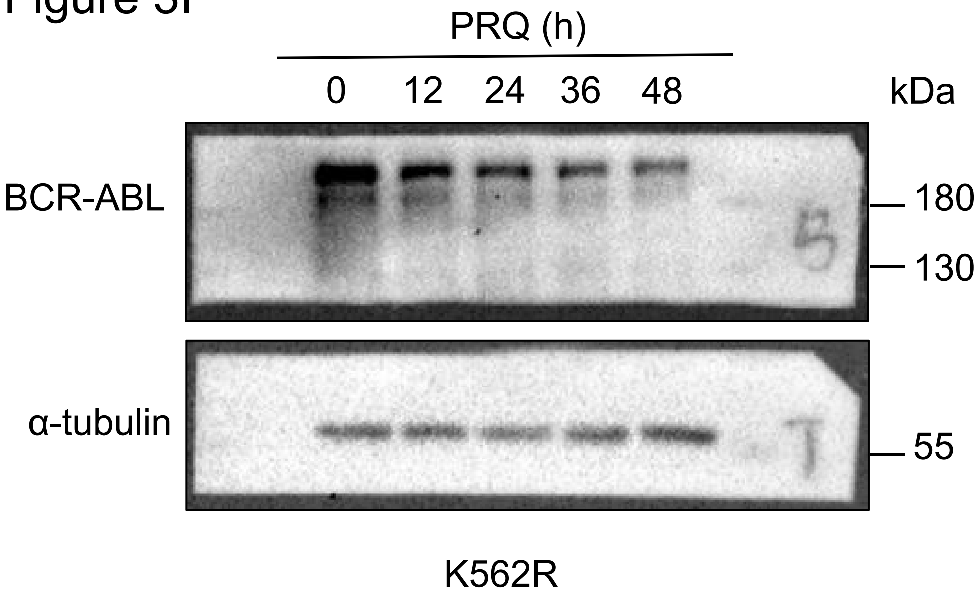

Figure 3N

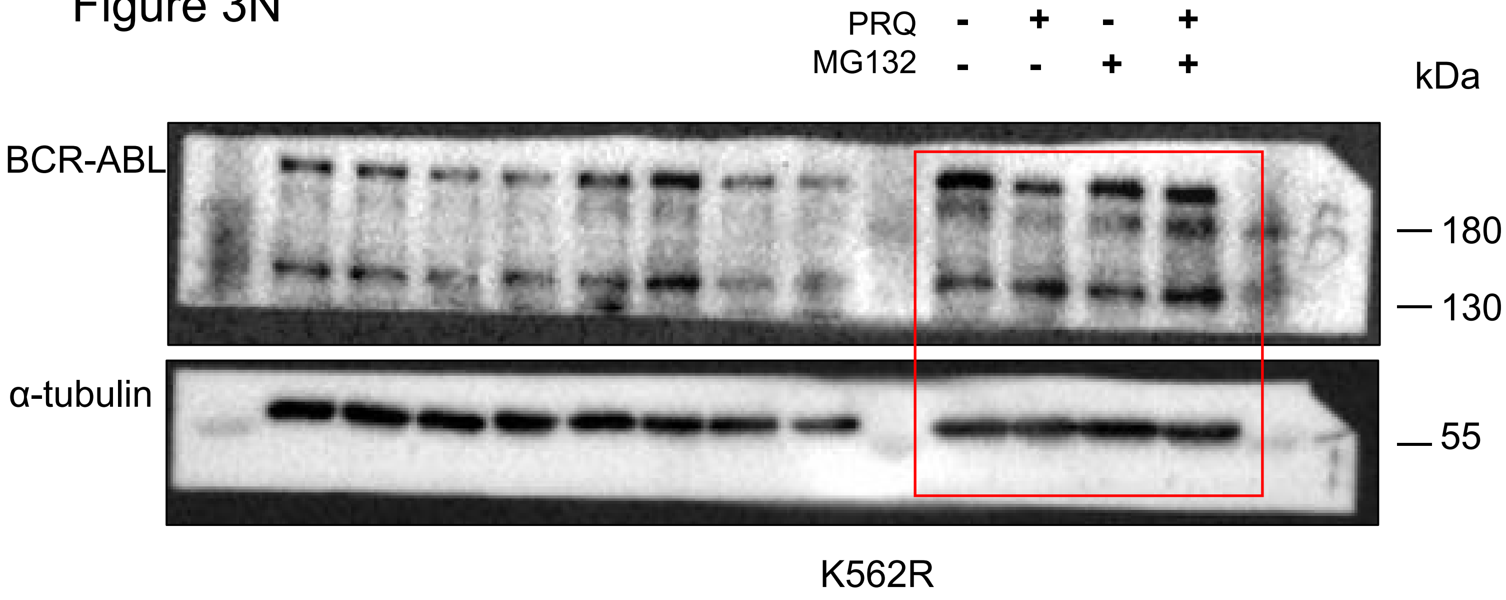

Figure 4

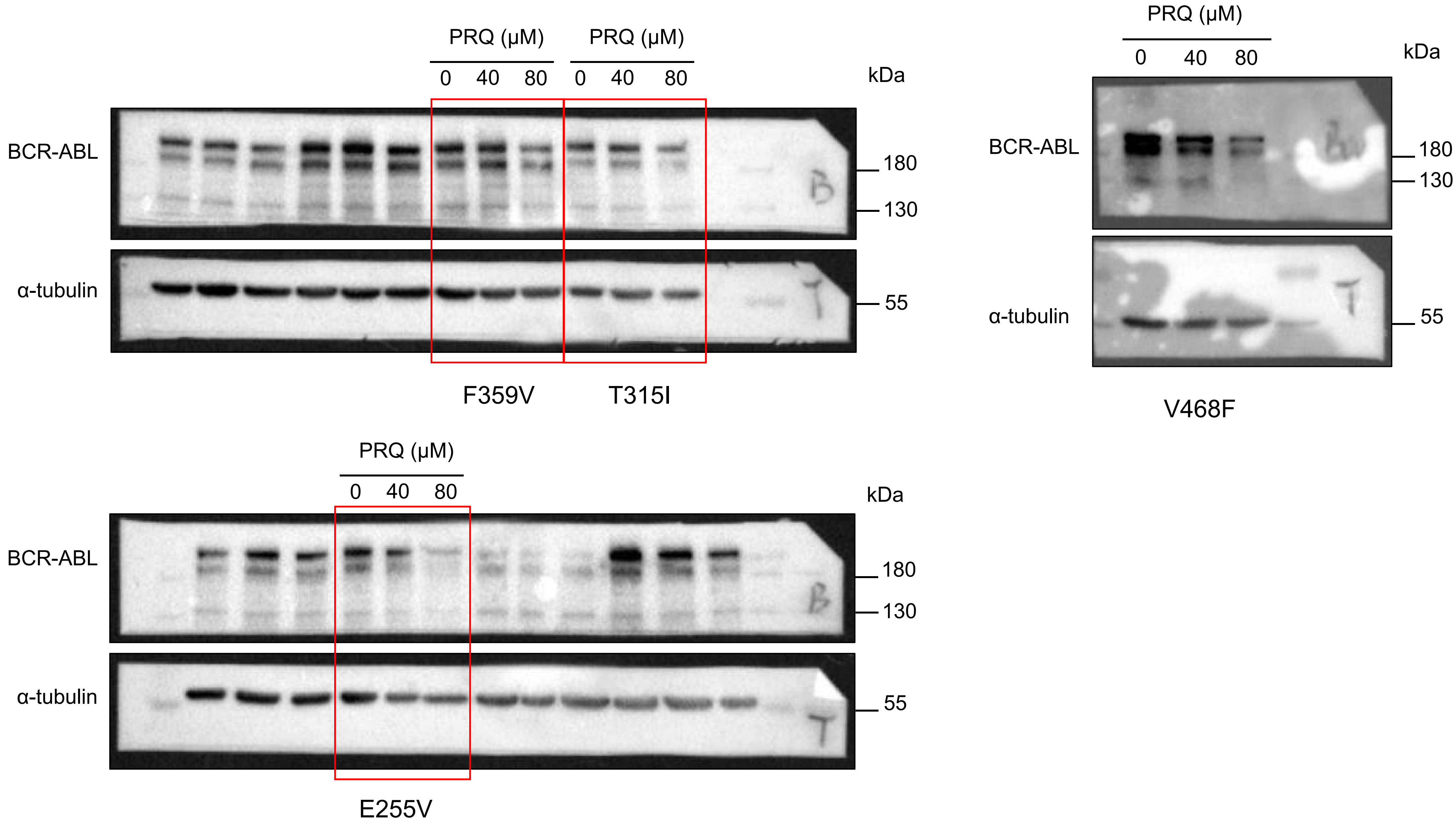
PRQ (μM)
0 40 80
PRQ (μM)
0 40 80
kDa
BCR-ABL
180
130
α-tubulin
55
F359V
T315I
PRQ (μM)
0 40 80
kDa
BCR-ABL
180
130
α-tubulin
55
V468F
PRQ (μM)
0 40 80
kDa
BCR-ABL
180
130
α-tubulin
55
E255V

Figure 6D

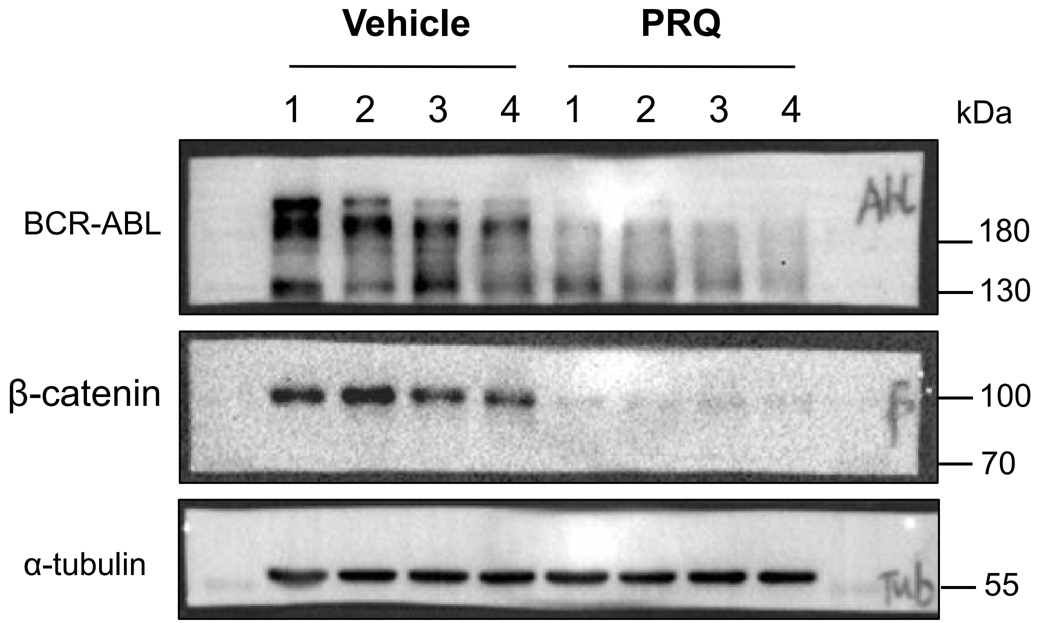

Vehicle
PRQ
1 2 3 4 1 2 3 4
kDa
BCR-ABL
180
130
β-catenin
100
70
α-tubulin
55

Figure S 1B

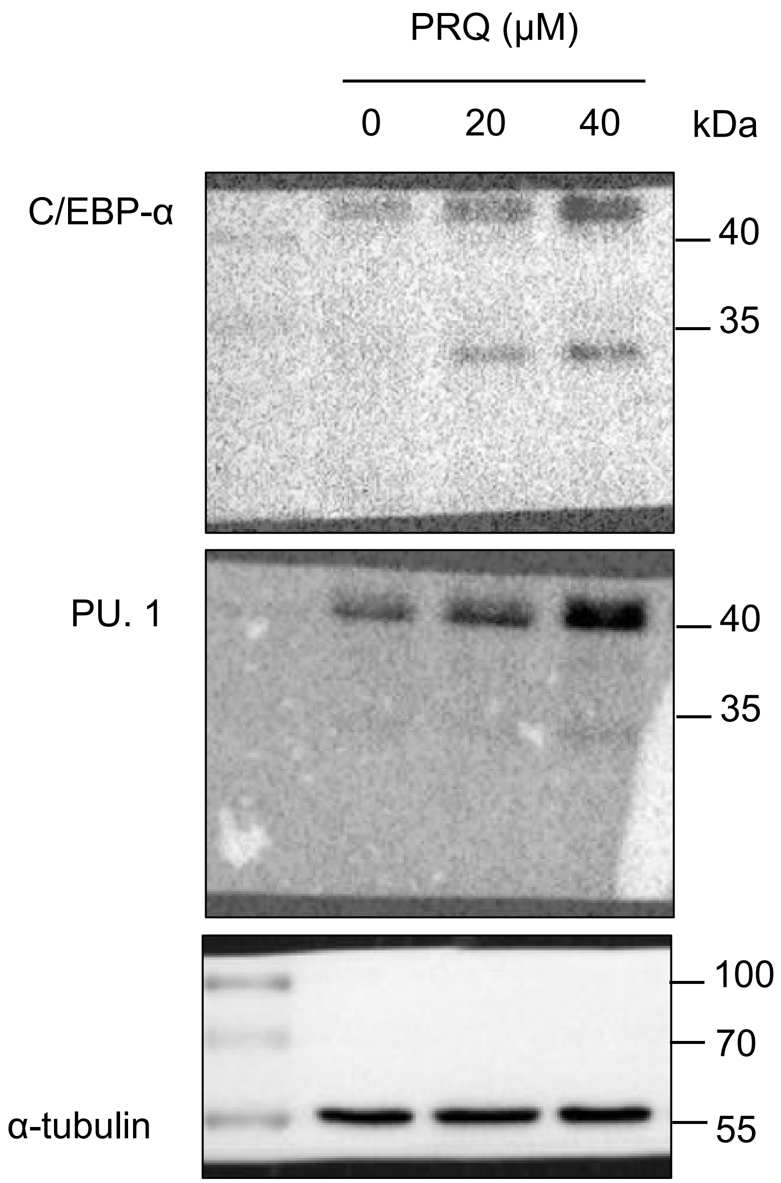

Figure S 3C

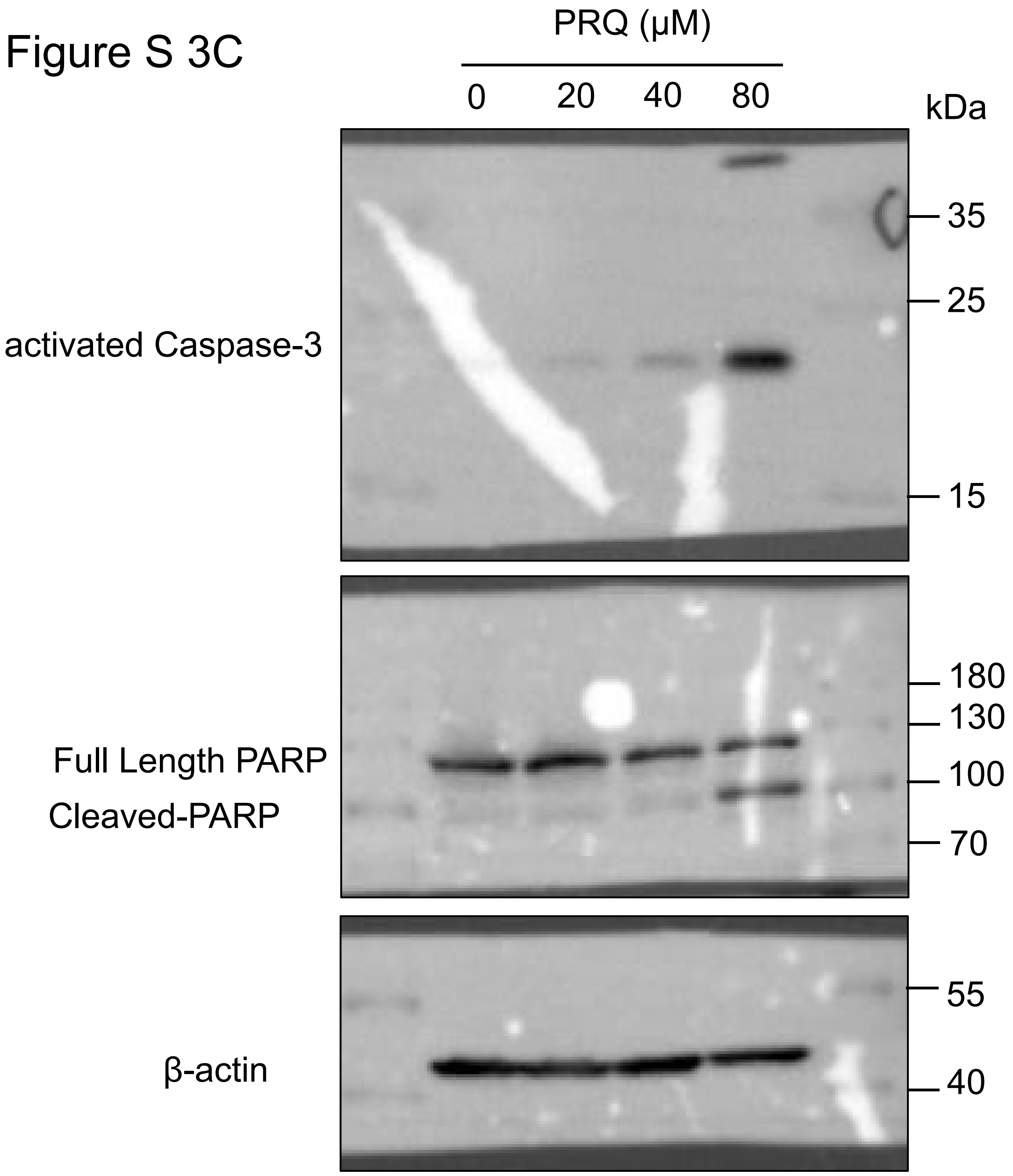

Figure S 4A

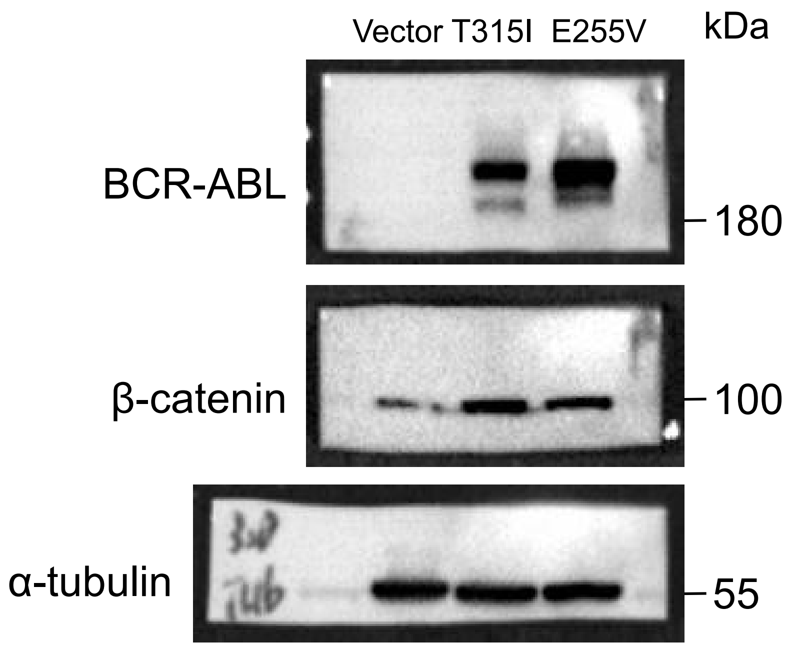

Figure S 4C

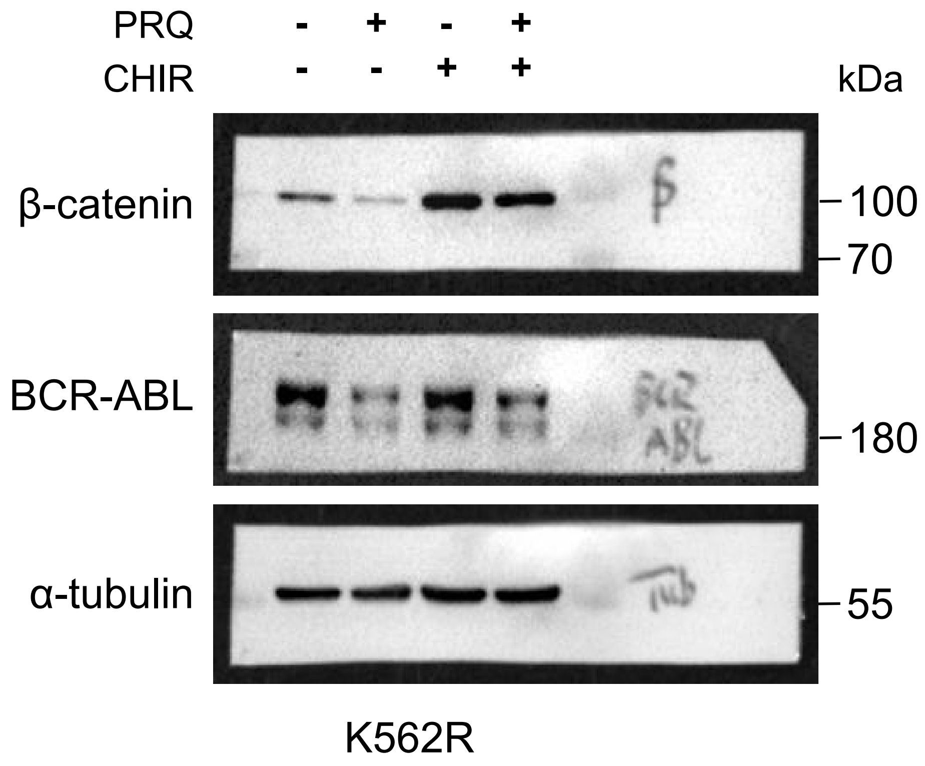

Figure S 5C

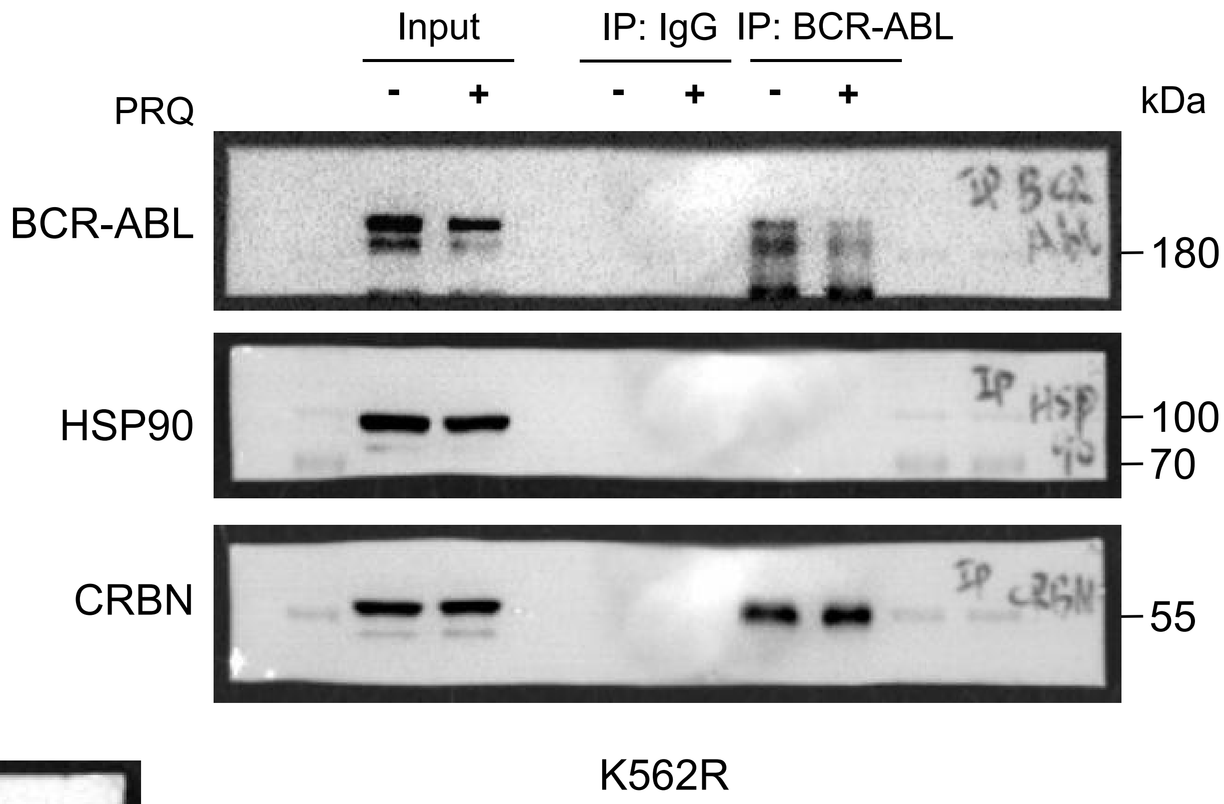

Figure S 5A

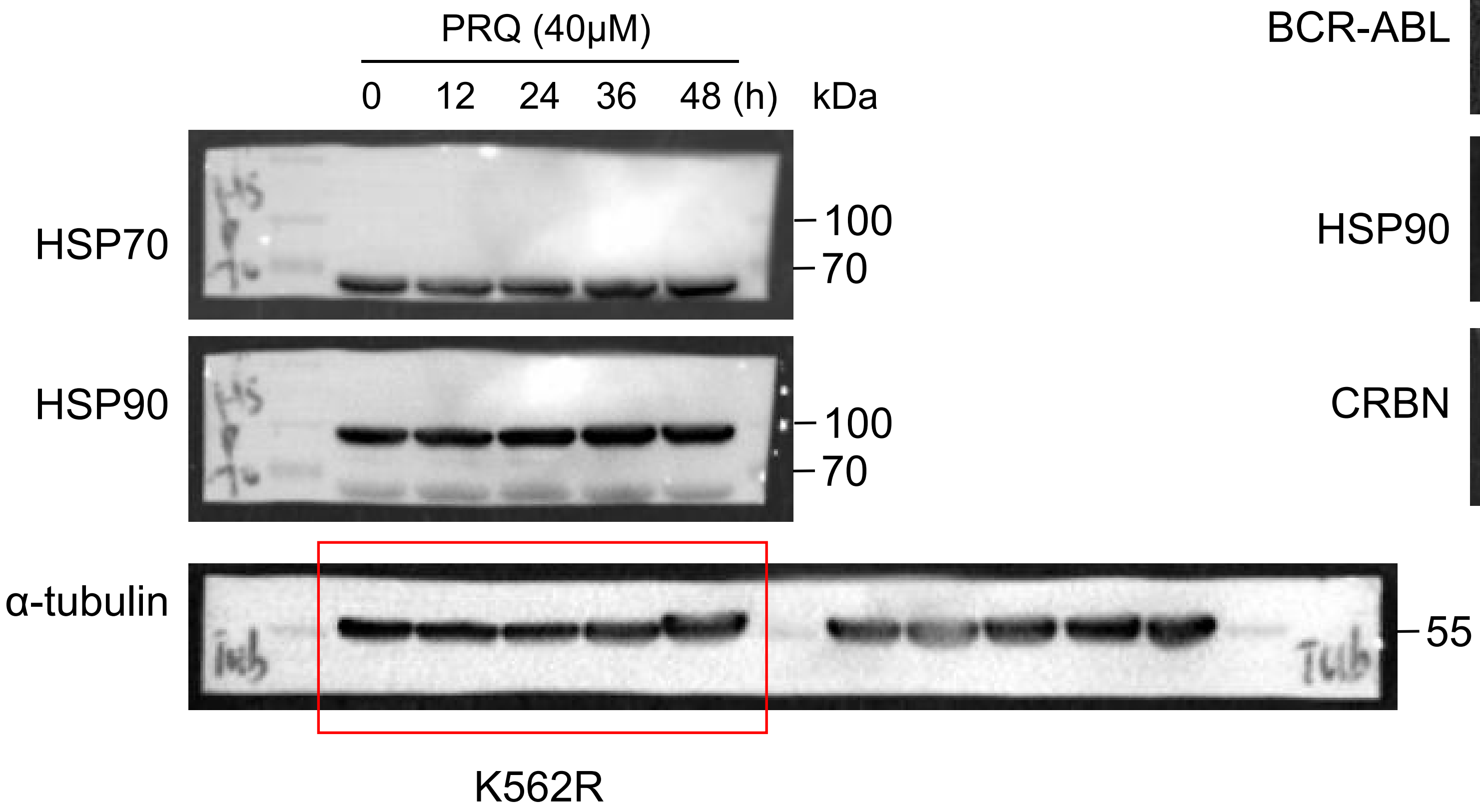

Supplement: Document S1. Figures S1–S7 and Tables S1 and S2 [file mmc1.pdf]
